# Mechanosensitive Piezo1/Osteocalcin/Irisin Axis Protects Against Disuse‐Induced Muscle Atrophy

**DOI:** 10.1002/advs.75355

**Published:** 2026-04-17

**Authors:** Zhaolu Wang, Xiuying Jiang, Xi Sun, Hao Chen, Jianjun Jin, Xin'e Shi

**Affiliations:** ^1^ College of Animal Science and Technology Northwest A&F University Yangling China

**Keywords:** Fndc5/Irisin, muscle atrophy, osteocalcin, Piezo1

## Abstract

Disuse‐induced muscle atrophy remains a therapeutic challenge due to its complex etiology. Osteocalcin (OCN) is a bone‐derived hormone with metabolic functions, while its role in muscle atrophy remains poorly understood. Here, we identified a mechanosensitive Piezo1/osteocalcin/Irisin axis linking bone mechanotransduction to muscle homeostasis. We showed that bilateral hindlimb immobilization (IMM) markedly reduced circulating undercarboxylated OCN. OCN deficiency exacerbated IMM‐induced muscle atrophy, whereas exogenous OCN attenuated muscle atrophy and promoted recovery. Mechanistically, Piezo1 acted as an upstream regulator of OCN, as pharmacological Piezo1 activation attenuated muscle atrophy in an OCN‐dependent manner, whereas bone‐specific Piezo1 knockdown abolished these protective effects. Furthermore, OCN exerted protective effects through the muscle receptor Gprc6a and the downstream effector Fndc5/Irisin, muscle‐specific knockdown of either Gprc6a or Fndc5 abolished OCN‐mediated protective effects. Notably, pair‐feeding experiments demonstrated that OCN directly protects against muscle atrophy independent of increased food intake. Finally, we demonstrated functional conservation of this axis in porcine myotubes. Notably, only animal models were used in the current study, and future studies are needed to test if the signaling axis has relevance to humans. Collectively, this work establishes that the Piezo1/Osteocalcin/Irisin axis mediates mechanical unloading‐induced muscle atrophy and highlights this axis as a promising therapeutic target for disuse‐induced muscle atrophy.

## Graphical abstract

Mechanical unloading suppresses bone Piezo1 expression, which reduces circulating undercarboxylated osteocalcin (unOCN). unOCN reduction subsequently exacerbates IMM‐induced Fndc5/Irisin decrease and drives severe muscle atrophy. Bone Piezo1 activation or exogenous osteocalcin/Irisin ameliorate muscle atrophy, while muscle‐specific Gprc6a or Fndc5 knockdown abolishes osteocalcin‐mediated protection.

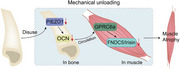

## 1 Introduction

Disuse, such as prolonged immobilization (IMM) [1], bed rest [2], or sedentary behavior [3], critically disrupts muscle mass, strength, and metabolic homeostasis by depriving mechanical loading. Mechanotransduction through mechanosensors, such as Piezo1, is critical for muscle homeostasis. Experimental disuse models demonstrate that Piezo1 inactivation during disuse correlates with elevated atrophy markers in muscle [1] and sclerostin‐driven osteopenia in bone [4, 5]. The musculoskeletal system relies on bidirectional mechanical and biochemical crosstalk, such as muscle‐derived loading cues sustaining bone density and bone‐secreted factors influencing muscle metabolism [6]. Emerging evidence suggests that bone‐derived factors play an important role in muscle metabolism and function [7, 8], while the role of bone‐derived endocrine signals in disuse‐induced muscle atrophy remains poorly understood.

Osteocalcin (OCN) is a bone‐derived hormone primarily secreted by osteoblasts that regulates a range of physiological processes, including increasing muscle glucose uptake [9], stimulating myoblast proliferation [10], maintaining muscle mass in older mice [11], and increasing exercise capacity [12]. These pleiotropic anabolic effects suggest that OCN may play a protective role against muscle atrophy. However, the OCN‐knockout (OCN^−/−^) mice models or OCN^−/−^ rat model challenge this potential that OCN^−/−^ mice/rat exhibit normal body weight, glucose level, bone mass, and muscle mass [13, 14, 15]. Furthermore, Lin et al. found that exogenous OCN treatment had minimal effects on muscle mass and whole‐body glucose handling in a unilateral hindlimb IMM mouse model [16].

Irisin, a myokine derived from the cleavage of fibronectin type III domain‐containing 5 (FNDC5), promotes skeletal muscle hypertrophy and ameliorates muscle wasting [17, 18]. Emerging evidence reveals striking functional parallels between OCN and Fndc5/Irisin. Both OCN and Fndc5/Irisin are upregulated in response to physical activity [9, 19] and favor glucose metabolism [9, 20]. Additionally, OCN and Fndc5/Irisin also play regulatory roles in bone [21, 22], adipose tissue [22, 23], liver [24, 25], pancreas [26, 27], and brain [28, 29]. This evidence indicates that OCN may modulate Fndc5/Irisin expression to influence muscle adaptation, whereas Fndc5/Irisin in turn regulates osteoblast activity. Understanding the OCN/Irisin axis may uncover a mechano‐hormonal pathway linking Piezo1‐mediated bone mechanotransduction to muscle homeostasis under disuse conditions.

In this study, we uncover a mechanosensitive Piezo1/Osteocalcin/Irisin axis that links mechanical unloading to muscle atrophy. Mechanical unloading suppresses skeletal Piezo1/osteocalcin signal, which in turn impairs Fndc5/Irisin signaling, thereby triggering disuse‐induced muscle atrophy. Skeletal Piezo1 activation or exogenous osteocalcin/Irisin administration attenuates muscle atrophy. Moreover, we demonstrated the functional conservation of the OCN/Irisin axis in pigs. Together, these findings identify the Piezo1/Osteocalcin/Irisin axis as a promising therapeutic target for disuse‐induced muscle atrophy.

## 2 Results

### 2.1 Exogenous OCN Alleviates IMM‐Induced Muscle Atrophy

**FIGURE 1:**
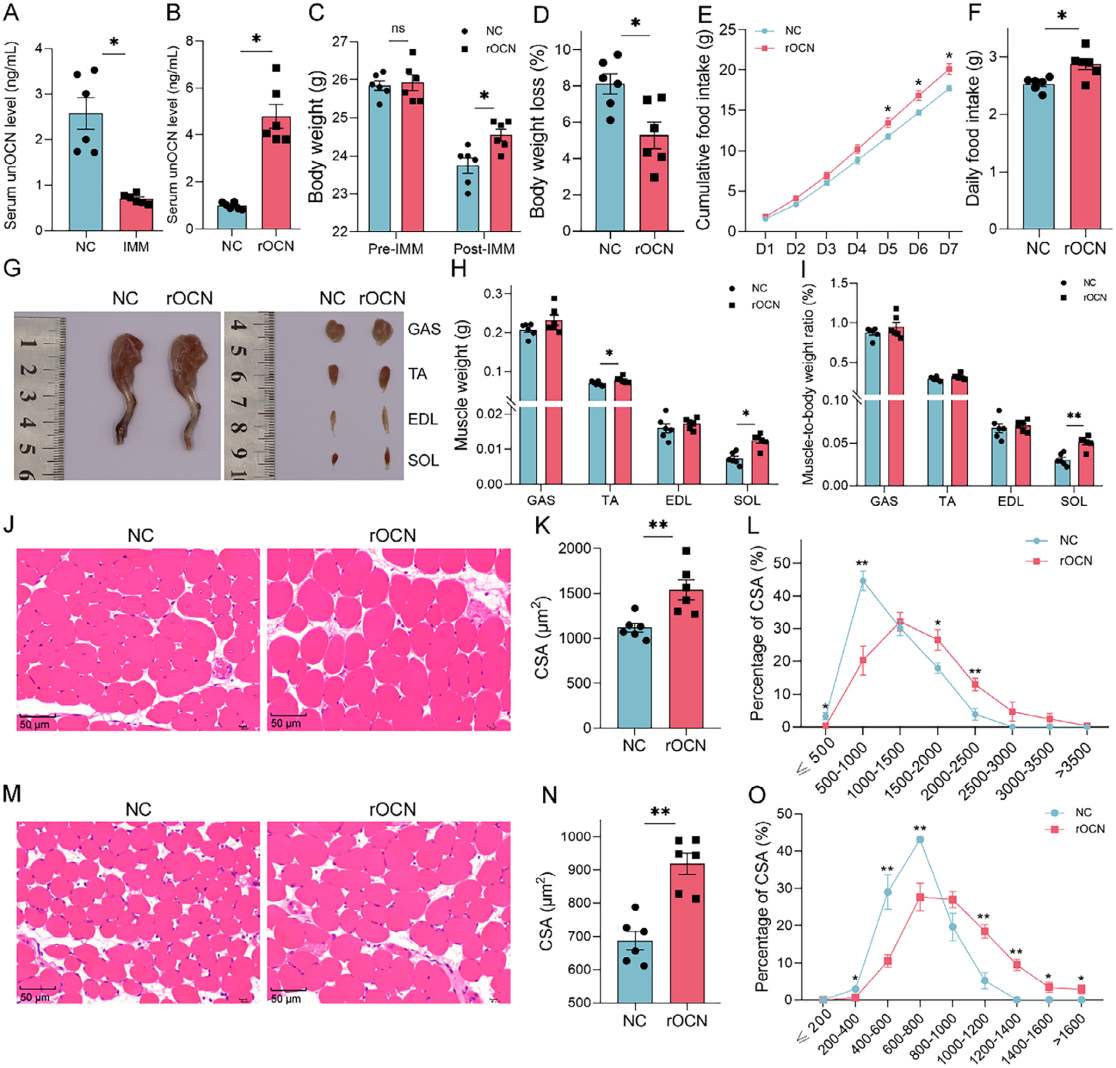
Exogenous OCN ameliorates IMM‐induced muscle atrophy. (A) Serum uncarboxylated osteocalcin (unOCN) levels from mice underwent seven days IMM. (B) Serum unOCN levels from IMM mice receiving intraperitoneal injection (IP) of rOCN. (C,D) Body weight changes (C) and body weight loss (D). (E,F) Quantification of cumulative food intake (E) and daily food intake (F). (G) Representative images of hindlimbs and GAS, TA, EDL, and SOL muscles. (H) Weight of GAS, TA, EDL, and SOL muscles. (I) Muscle‐to‐body weight ratios of GAS, TA, EDL and SOL. (J–L) Representative H&E staining (J), mean myofiber CSA (K) and percentage of CSA distribution (L) of TA muscles. (M‐O) Representative H&E staining (M), mean myofiber CSA (N) and percentage of CSA distribution (O) of SOL muscles. Age and body weight‐matched male adult WT mice were used to generate IMM‐induced muscle atrophy model, the rOCN was injected intraperitoneally at 30 ng/g body weight daily. Samples were collected at day 7 post‐IMM. Representative images (scale bar = 50 µm) captured at 400× magnification. n = 6 for each group unless otherwise specified, data points show individual mice. Data are represented as mean ± SEM and were analyzed by unpaired Two‐tailed Student's *t* tests. ^*^ *p* <0.05.^**^ *p* <0.01.

To determine whether OCN is involved in IMM‐induced muscle atrophy, we established a seven‐day bilateral hindlimb IMM model in wild‐type (WT) mice, which caused ∼10% body weight loss (Figure S1A,B), and significant reductions in cumulative and daily food intake (Figure S1C,D). IMM also decreased muscle weight and muscle‐to‐body weight ratios of gastrocnemius (GAS), tibialis anterior (TA), extensor digitorum longus (EDL), and soleus (SOL) (Figure S1E,G), and the cross‐sectional area (CSA) in both fast‐twitch muscle (TA) (Figure S1H–J) and slow‐twitch muscle (SOL) (Figure S1K–M). Notably, serum undercarboxylated OCN (unOCN) levels exhibited a ∼70% decrease (Figure 1A), suggesting OCN is associated with IMM‐induced muscle atrophy.

We next investigated whether exogenous OCN alleviated muscle atrophy. Daily intraperitoneal injection (IP) of recombinant OCN protein (rOCN) significantly increased serum unOCN level in IMM mice (Figure 1B), and alleviated IMM‐induced body weight loss (Figure 1C,D). The rOCN‐treated mice also maintained higher cumulative food intake (Figure 1E) and daily food intake (Figure 1F). Moreover, rOCN significantly alleviated IMM‐induced muscle weight decreases in TA and SOL muscles (Figure 1G,H), and the muscle‐to‐body weight ratio of SOL (Figure 1I). Besides, rOCN mitigated IMM‐induced reductions of myofiber CSA and led to a rightward (larger) shift in CSA distribution of both TA (Figure 1J–L) and SOL (Figure 1M–O). The results demonstrated that OCN alleviates IMM‐induced muscle atrophy.

Under normal physiological conditions (non‐atrophy), rOCN administration (Figure S2A) significantly promoted exercise capacity (Figure S2B,C), whereas it did not induce observable changes in body weight (Figure S2D), food intake (Figure S2E,F), muscle weight (Figure S2G,H), muscle‐to‐body weight ratio (Figure S2I), or myofiber CSA (Figure S2J–O). Collectively, these findings suggest that OCN alleviates IMM‐induced muscle atrophy.

### 2.2 OCN Deficiency Aggravates IMM‐Induced Muscle Atrophy

To further determine the effects of OCN in skeletal muscle, we generated global osteocalcin knockout mice (Figure S3A,B) and compared muscle phenotypes among littermate WT, heterozygous (OCN^+/−^), and homozygous (OCN^−/−^) mice. No significant differences were observed in body weight (from 4 to 12 weeks of age) (Figure S3C) and food intake (at 12 weeks of age) (Figure S3D) across genotypes. However, the OCN^−/−^ mice, not OCN^+/−^mice, exhibited significant decreases of maximum speed (Figure S3E) and running distance (Figure S3F) compared to WT littermates. Notably, indirect calorimetry analysis between WT and OCN^−/−^ littermates demonstrated the altered respiratory exchange ratio (RER), with OCN^−/−^ mice showing a higher late‐night, not the whole night, RER (Figure S3G,H), indicating the decreased fatty acid oxidation. However, the heat production remained unchanged (Figure S3I,J). No observable changes were detected in muscle weight (Figure S3K,L), muscle‐to‐body weight ratio (Figure S3M), the CSA of TA (Figure S3N–P) and SOL (Figure S3Q–S). Collectively, OCN deficiency impairs exercise performance and RER, but does not induce muscle atrophy.

**FIGURE 2:**
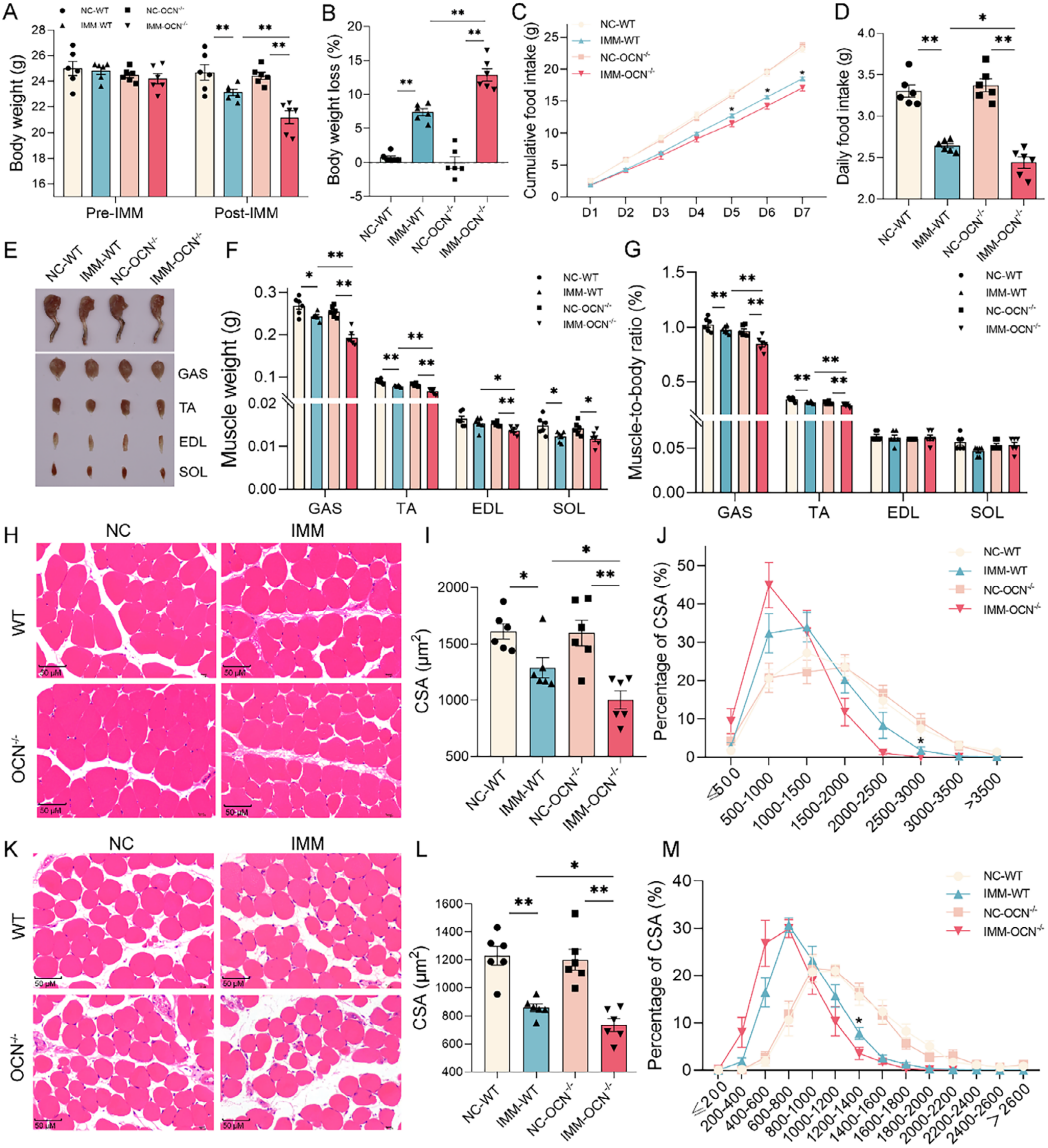
OCN deficiency aggravates IMM‐induced muscle atrophy. (A,B) Body weight changes (A) and body weight loss (B) of mice underwent seven days IMM. (C,D) Quantification of cumulative food intake (C) and daily food intake (D). (E) Representative images of hindlimbs and GAS, TA, EDL, and SOL muscles. (F) Weight of GAS, TA, EDL, and SOL muscles. (G) Muscle‐to‐body weight ratios of GAS, TA, EDL and SOL. (H–J) Representative H&E staining (H), mean myofiber CSA (I) and percentage of CSA distribution (J) of TA muscles. (K‐M) Representative H&E staining (K), mean myofiber CSA (L) and percentage of CSA distribution (M) of SOL muscles. Age and body weight‐matched male adult WT and littermates OCN^−/−^ mice were used. Samples were collected at day seven post‐IMM. Representative images (scale bar = 50 µm) captured at 400× magnification. n = 6 for each group unless otherwise specified, data points show individual mice. Data are represented as mean ± SEM and were analyzed by two‐way ANOVA followed by Sidak's multiple comparisons test for pre‐selected comparisons. ^*^ *p* <0.05.^**^ *p* <0.01. The ^*^ in Figure C, J, M mean significant difference between IMM‐WT vs IMM‐OCN^−/−^.

To further evaluate the effects of OCN on muscle atrophy, we generated IMM‐induced muscle atrophy in WT mice (NC‐WT vs. IMM‐WT) and OCN^−/−^ littermates (NC‐OCN^−/−^vs. IMM‐OCN^−/−^). The IMM‐OCN^−/−^ mice exhibited more severe body weight loss compared to their IMM‐WT littermates (Figure 2A,B), accompanied by significant reductions of food intake (Figure 2C,D). Notably, muscle weights of GAS, TA, and EDL (Figure 2E,F) and muscle‐to‐body weight ratios of GAS and TA (Figure 2G) were significantly decreased in IMM‐OCN^−/−^ mice compared with IMM‐WT littermates. Moreover, IMM‐OCN^−/−^ showed observable decreases of myofiber CSA and leftward (smaller) shift of CSA distributions in both TA (Figure 2H–J) and SOL (Figure 2K–M) compared with IMM‐WT littermates. These data collectively demonstrate that OCN deficiency aggravates IMM‐induced muscle atrophy.

### 2.3 Exogenous OCN Ameliorates IMM‐Induced Muscle Atrophy and Promotes the Recovery of Atrophied Muscles

The beneficial effects of OCN have consistently been associated with increased food intake. To determine whether its protection against muscle atrophy is primarily a direct action on muscle or an indirect effect mediated through enhanced food intake, we performed a pair‐feeding experiment in age‐ and weight‐matched male OCN^−/−^ mice subjected to IMM. Mice were assigned to three groups: vehicle‐treated with ad libitum feeding (NC‐Ad libitum), rOCN‐treated with food intake pair‐fed to the Vehicle Ad lib group (rOCN‐Pair fed), and rOCN‐treated with ad libitum feeding (rOCN‐Ad libitum). Critically, even when food intake was restricted to the level of NC‐Ad libitum mice (Figure S4C,D), rOCN administration significantly attenuated IMM‐induced muscle atrophy. Compared to the NC‐Ad libitum mice, the rOCN‐Pair fed mice exhibited reduced body weight loss (Figure S4A,B), preserved muscle mass (Figure S4E–G), and increased mean CSA with a rightward (larger) shift in CSA distribution in both TA (Figure S4H–J) and SOL (Figure S4K–M) muscles. This result demonstrated a direct protective effect of OCN against muscle atrophy. Meanwhile, rOCN administration in ad libitum‐fed mice (rOCN‐Ad libitum) also significantly protected against muscle atrophy and significantly increased food intake (Figure S4). Notably, TA weight was significantly higher in the rOCN‐Ad libitum mice than it in the rOCN‐Pair fed mice (Figure S4E,F), indicating that the orexigenic effect of OCN provides an additional, synergistic benefit to muscle preservation.

**FIGURE 3:**
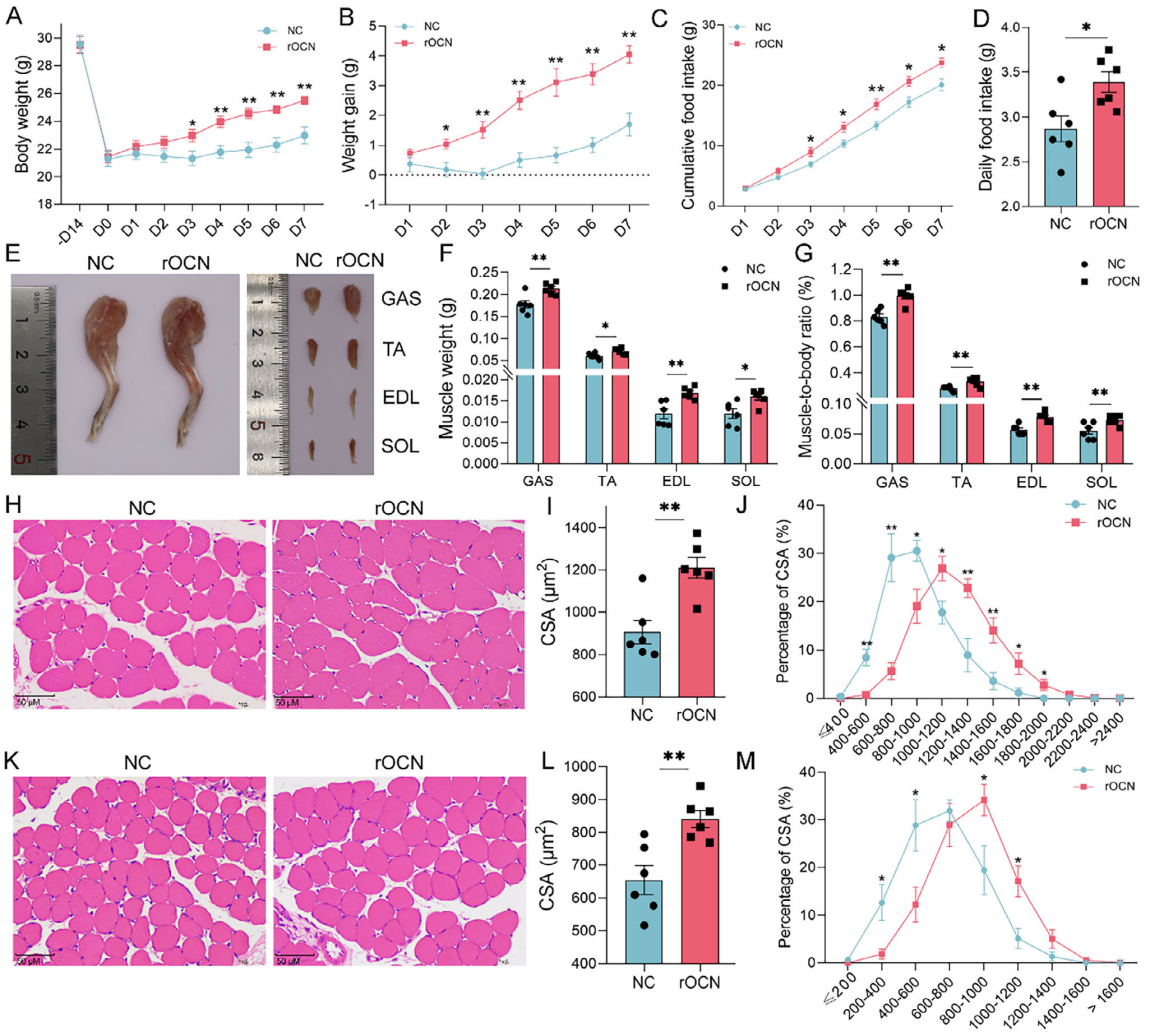
Exogenous OCN promotes the recovery of atrophied muscles in OCN^−/−^ mice. (A,B) Body weight changes of mice during IMM (‐D14‐D0) and recovery (D0‐D7) (A) and weight gains from D0 to D7 (B). (C,D) Quantification of cumulative food intake (C) and daily food intake (D). (E) Representative images of hindlimbs and GAS, TA, EDL, and SOL muscles. (F) Weight of GAS, TA, EDL, and SOL muscles. (G) Muscle‐to‐body weight ratios of GAS, TA, EDL and SOL. (H‐J) Representative H&E staining (H), mean myofiber CSA (I) and percentage of CSA distribution (J) of TA muscles. (K‐M) Representative H&E staining (K), mean myofiber CSA (L) and percentage of CSA distribution (M) of SOL muscles. Age and body weight‐matched male adult OCN^−/−^ mice underwent 14 days of bilateral hindlimb IMM to induce severe muscle atrophy, followed by removal of the IMM (Defined as Day 0, D0). Mice with similar body weight before and after IMM were randomly assigned to group receiving rOCN administration or control. The rOCN was injected intraperitoneally at 30 ng/g body weight daily. Samples were collected at day seven post‐removal of IMM. Representative images (scale bar = 50 µm) captured at 400× magnification. n = 6 for each group unless otherwise specified, data points show individual mice. Data are represented as mean ± SEM and were analyzed by unpaired Two‐tailed Student's t tests. ^*^ *p* <0.05.^**^ *p* <0.01.

Having established the direct anti‐atrophic action of OCN, we next assessed the therapeutic potential of OCN in atrophic muscle recovery. OCN^−/−^ mice were subjected to a 14‐day IMM to induce severe muscle atrophy, and then received either rOCN administration or vehicle control for seven days (Figure 3A). rOCN significantly enhanced recovery of mice, as indicated by higher body weight (Figure 3A) and body weight gain (Figure 3B). Besides, rOCN also significantly increased cumulative food intake (Figure 3C) and daily food intake (Figure 3D). Notably, rOCN significantly increased muscle weight and muscle‐to‐body weight ratios of GAS, TA, EDL, and SOL (Figure 3E–G), myofiber CSA, and led to a rightward (larger) shift of CSA distributions of both TA (Figure 3H–J) and SOL (Figure 3K–M).

Although significant body weight gain in OCN^−/−^ mice was observed as early as day 2 with rOCN treatment, a parallel experiment in WT mice revealed that rOCN requires a prolonged duration to elicit observable benefits. Specifically, rOCN administration significantly increased food intake (Figure S5C,D), while seven‐day rOCN administration did not induce significant effect in body weight, a longer term (14d) was required to elicit significant increases in body weight (Figure S5A) and body weight gain (Figure S5B), muscle weight in GAS, TA and SOL (Figure S5E,F), muscle‐to‐body weight ratios of TA and SOL (Figure S5G), and myofiber CSA (Figure S5H–M). Collectively, our results demonstrated that OCN exerts a direct protective effect against muscle atrophy and promotes the recovery of atrophied muscle.

### 2.4 Pharmacological Activation of Skeletal Piezo1 Alleviates IMM‐Induced Muscle Atrophy Through OCN

**FIGURE 4:**
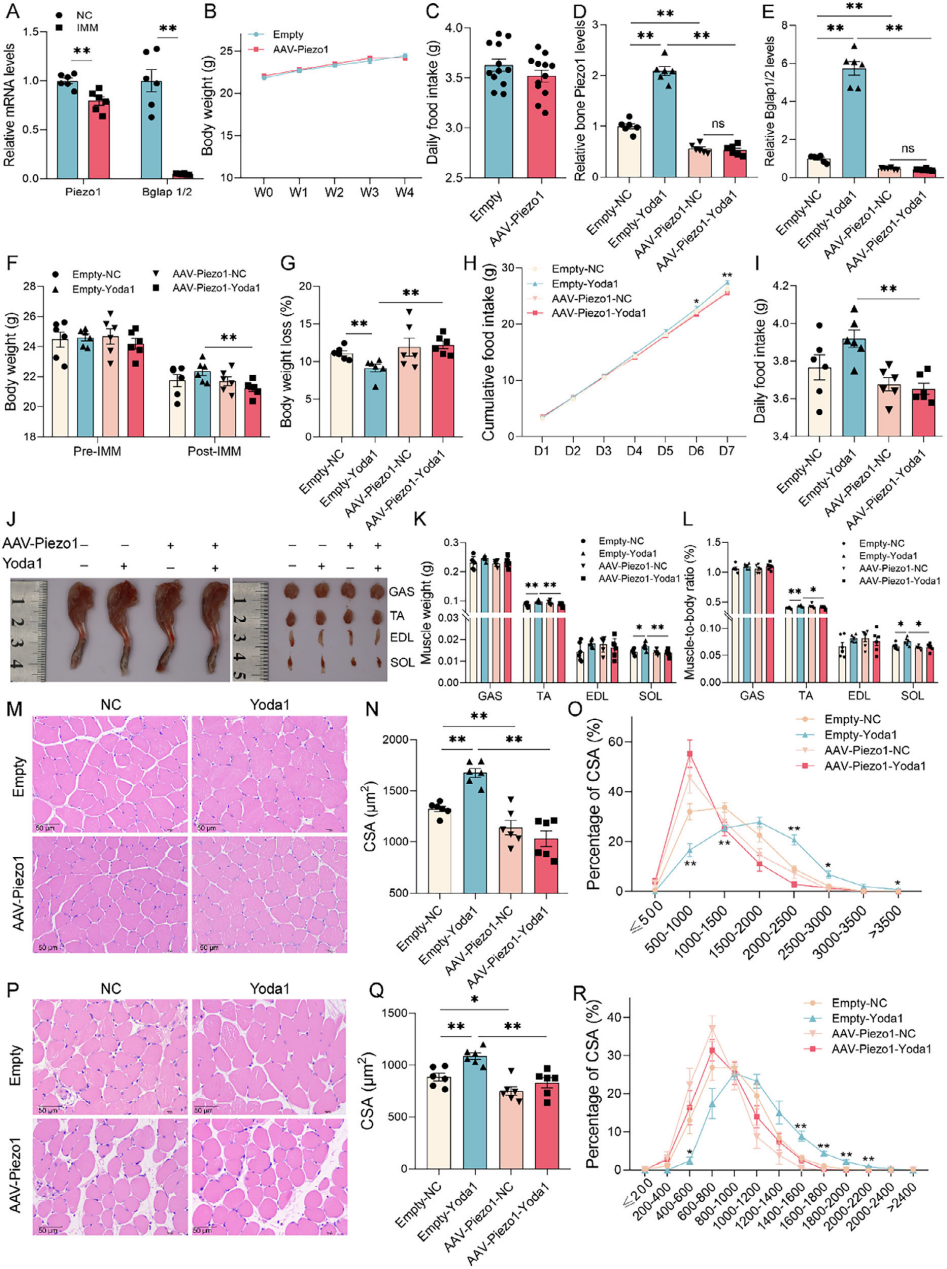
Pharmacological Piezo1 activation alleviates muscle atrophy in WT mice. (A) Relative mRNA levels of bone Piezo1 and Bglap1/2 of mice underwent seven‐day IMM, Gapdh was used as reference gene (B) Body weight change after AAV‐Piezo1 injection into both tibias (n = 12). (C) Daily food intake at 4 weeks post‐AAV‐Piezo1 injection (n = 12). (D,E) Relative mRNA levels of Piezo1 (D) and Bglap1/2 (E) in tibia under IMM receiving daily Yoda1 administration for seven days, Gapdh was used as reference gene. (F,G) Body weight change (F) and body weight loss (G). (H,I) Quantification of cumulative food intake (H) and daily food intake (I). (J) Representative images of hindlimbs and GAS, TA, EDL, and SOL muscles. (K,L) Muscle weight (K) and muscle‐to‐body weight ratios (L) of GAS, TA, EDL and SOL muscles. (M–O) Representative H&E staining (M), mean myofiber CSA (N) and the percentage of CSA distribution (O) of TA muscles. (P‐R) Representative H&E staining (P), mean myofiber CSA (Q) and the percentage of CSA distribution (R) of SOL muscles. Body weight‐matched male WT mice at age of 8 week were used. AAV‐Piezo1 or the empty vector was injected via intramedullary route into bilateral tibias. IMM‐induced muscle atrophy model was generated at 5 weeks post‐AAV‐Piezo1 injection. The Yoda1 was injected intraperitoneally at dose of 0.2 mg/kg body weight daily during IMM. Samples were collected at day seven post‐IMM. Representative images (scale bar = 50 µm) captured at 400× magnification. n = 6 for each group unless otherwise specified, data points show individual mice. Data are represented as mean ± SEM and were analyzed by unpaired Two‐tailed Student's t tests and two‐way ANOVA followed by Sidak's multiple comparisons test for pre‐selected comparisons. ^*^ *p* <0.05.^**^ *p* < 0.01. The ^*^ in Figure G mean significant difference between Empty‐Yoda1 vs AAV‐Piezo1‐Yoda1, while the * in Figure N, Q mean significant difference between Empty‐NC vs Empty‐Yoda1.

We then evaluated the underlying mechanism by which IMM downregulated OCN level. Generally, the unOCN levels depend on both osteoblast synthesis (cell number/activity) and osteoclast‐mediated decarboxylation. We quantified osteoblast and osteoclast populations, but found no statistical difference between NC and IMM mice (Figure S6A–D), ruling out the altered cell numbers as a regulator of OCN decrease. We next investigated potential transcriptional regulators/pathways using a publicly available hindlimb unloading (HLU) transcriptomic dataset (GSE235942) (Figure S6E). The cytoskeletal (actinin binding), metabolic (amino acid transport), and inflammatory pathways (cytokine interaction) were significantly enriched, suggesting mechanical and metabolic dysregulation during HLU. Notably, GO analysis revealed significant enrichment in ion channel‐related terms (channel activity, passive transmembrane transport), alongside KEGG analysis, which revealed significant enrichment in calcium signaling (Figure S6F,G), highlighting the potential role of ion channel in HLU. Piezo1, a mechanosensitive ion channel, showed reduced expression in bone in IMM model (Figure 4A). We therefore hypothesized that skeletal Piezo1 was the upstream regulator of OCN in IMM model, and pharmacological activation of skeletal Piezo1 ameliorated muscle atrophy via upregulating OCN. We knockdown the skeletal Piezo1 via intramedullary injection of AAV‐Piezo1 into the tibia of wild‐type mice, and observed no statistical changes in body weight (Figure 4B) and food intake (Figure 4C) in the physiological state within 4 weeks post‐injection. Then we established IMM‐induced muscle atrophy model, and found that AAV‐Piezo1 significantly decreased skeletal Piezo1 level and abolished Yoda1 (a Piezo1 agonist)‐mediated skeletal Piezo1 upregulation (Figure 4D), while no statistical changes were observed in muscle Piezo1 (Figure S6H). Notably, Yoda1 administration effectively ameliorated IMM‐induced body weight loss in Empty mice, while this effect was abolished in AAV‐Piezo1 mice (Figure 4F,G). Notably, no significant changes in food intake were observed in either Empty or AAV‐Piezo1 mice following Yoda1 treatment compared to their respective controls. However, a direct comparison revealed that food intake was significantly higher in Empty‐Yoda1 mice than in AAV‐Piezo1‐Yoda1 mice (Figure 4H,I). Muscle analysis further revealed that Yoda1 administration increased muscle weight and muscle‐to‐body weight ratio of TA and SOL in Empty mice, while this protective effect was not observed in the AAV‐Piezo1 mice (Figure 4J–L). Moreover, Yoda1 administration significantly increased myofiber CSA and led a rightward (larger) shift of CSA distributions in both TA and SOL of Empty mice, while AAV‐Piezo1 abolished Yoda1‐mediated protective effects (Figure 4M–R).

**FIGURE 5:**
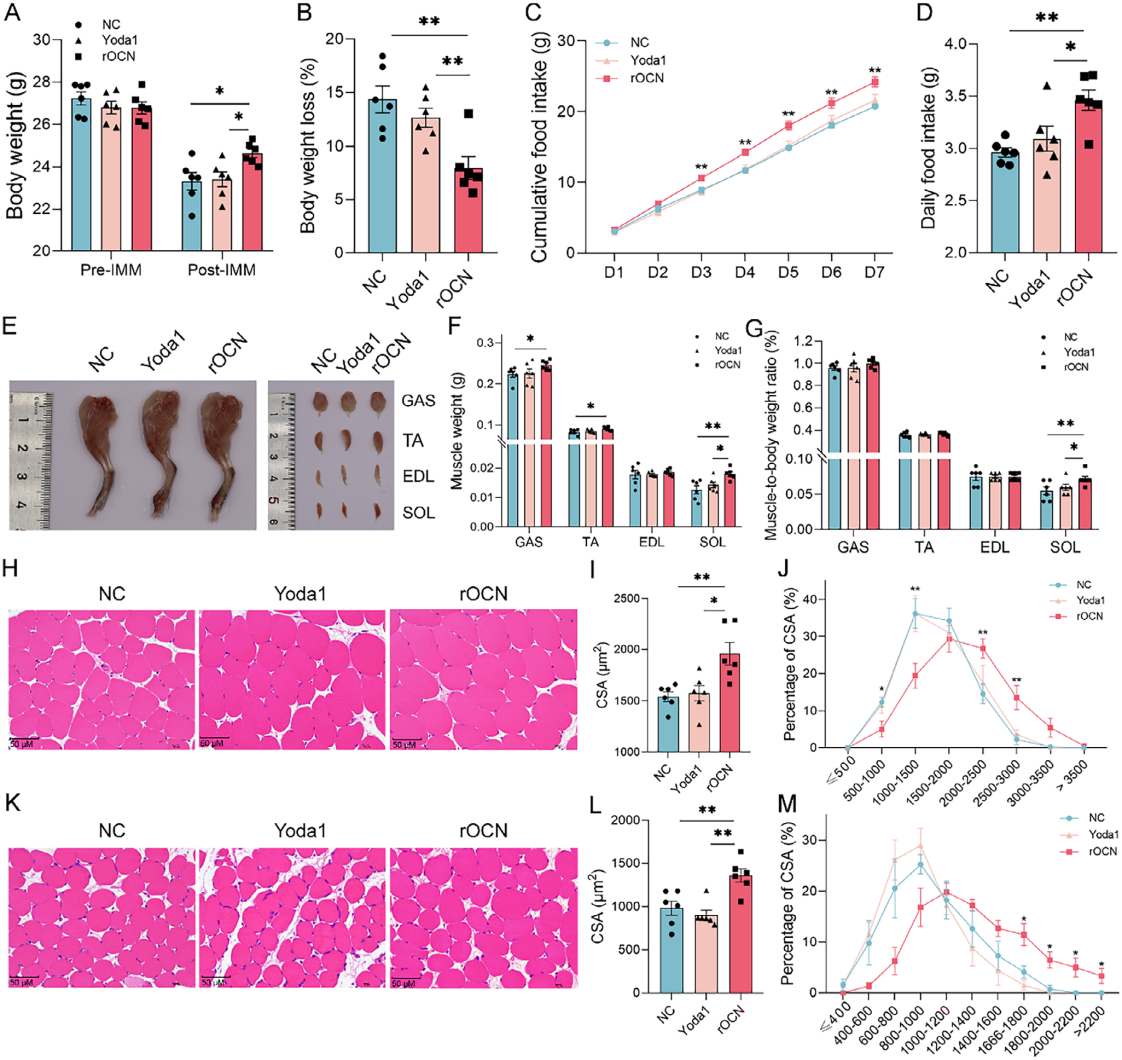
Piezo1 fails to protect against IMM‐induced muscle atrophy in OCN^−/−^ mice. (A,B) Body weight change (A) and body weight loss (B). (C,D) Quantification of cumulative food intake (C) and daily food intake (D). (E) Representative images of hindlimbs and GAS, TA, EDL, and SOL muscles. (F) Weight of GAS, TA, EDL, and SOL muscles. (G) Muscle‐to‐body weight ratios of GAS, TA, EDL and SOL muscles. (H–J) Representative H&E staining (H), mean myofiber CSA (I) and the percentage of CSA distribution (J) of TA muscles. (K–M) Representative H&E staining (K), mean myofiber CSA (L) and the percentage of CSA distribution (M) of SOL muscles. Age and body weight‐matched male adult OCN^−/−^ mice were used to generate IMM‐induced muscle atrophy model. The Yoda1 was injected intraperitoneally at dose of 0.2 mg/kg body weight daily, and the rOCN was injected intraperitoneally at 30 ng/g body weight daily. Samples were collected at day 7 post‐IMM. Representative images (scale bar = 50 µm) captured at 400× magnification. n = 6 for each group unless otherwise specified, data points show individual mice. Data are represented as mean ± SEM and were analyzed by one‐way ANOVA with Dunnett's multiple comparisons test. ^*^ *p* <0.05.^**^ *p* <0.01. The ^*^ in Figure C, J, M mean significant difference between NC vs rOCN.

Notably, the Bglap1/2 genes, which encode OCN, were markedly decreased in the bone of IMM mice (Figure 4A). Conversely, Yoda1 treatment significantly increased skeletal Bglap1/2 level in Empty mice, but not in AAV‐Piezo1 mice (Figure 4E), indicating a regulatory link between Piezo1 and OCN. To verify whether Piezo1 alleviated muscle atrophy in OCN‐dependent manner, we established IMM‐induced muscle atrophy model in OCN^−/−^ mice. Yoda1 administration failed to protect against IMM‐induced body weight loss (Figure 5A,B), food intake (Figure 5C,D), muscle loss (Figure 5E–G), or CSA reduction in either TA (Figure 5H–J) or SOL (Figure 5K–M). In contrast, rOCN effectively alleviated those muscle atrophy‐related phenotypes. Collectively, our findings in AAV‐Piezo1 mice and OCN^−/−^ mice collectively demonstrate that pharmacological activation of skeletal Piezo1 protects against disuse‐induced muscle atrophy in an OCN‐dependent manner.

### 2.5 Gprc6a is the Essential Receptor of OCN in Ameliorating IMM‐Induced Muscle Atrophy

**FIGURE 6:**
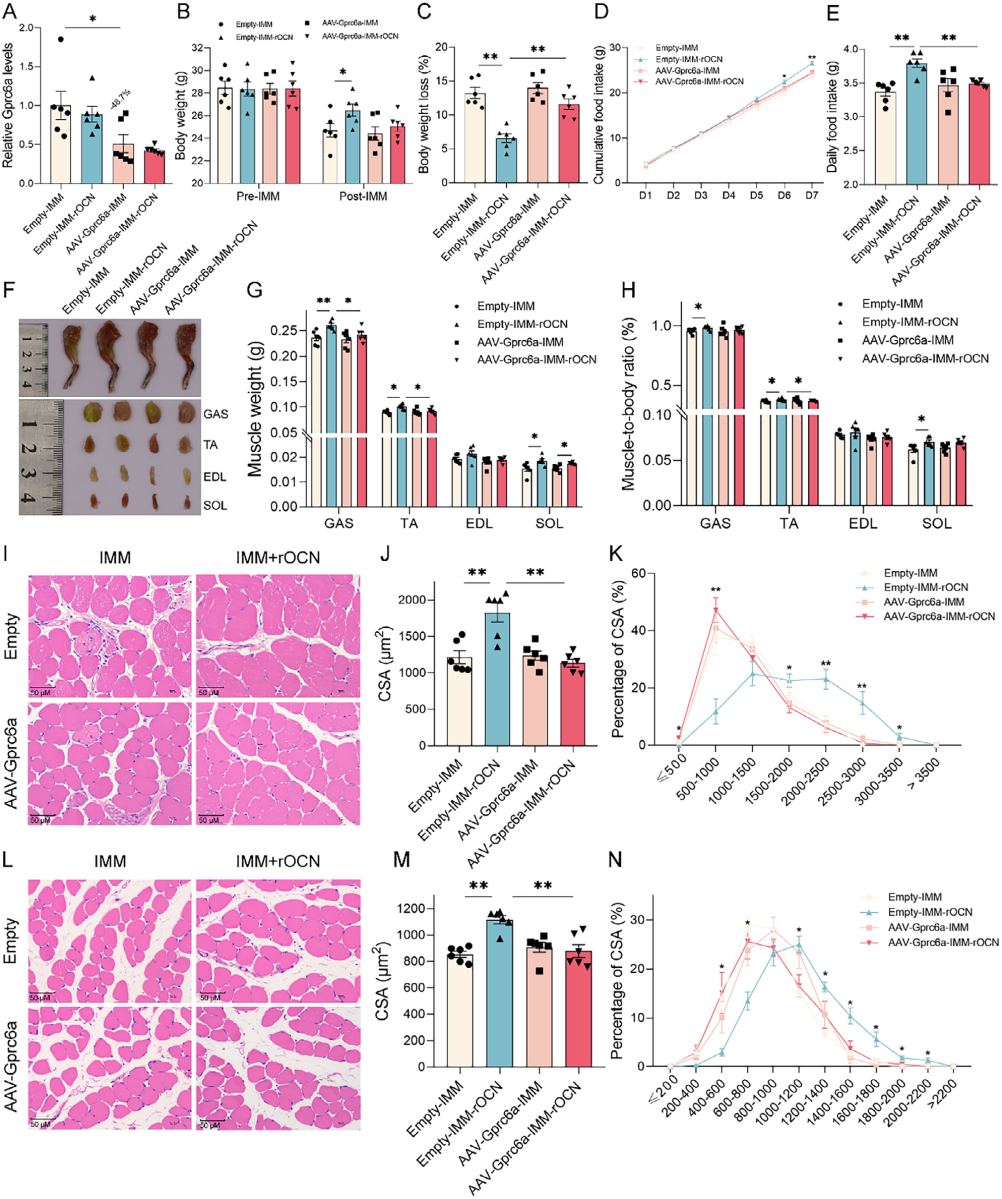
Gprc6a is required in OCN‐induced protective effects. (A) Relative mRNA levels of muscle Gprc6a, Gapdh was used as reference gene. (B,C) Body weight change (B) and body weight loss (C). (D,E) Quantification of cumulative food intake (D) and daily food intake (E). (F) Representative images of hindlimbs and GAS, TA, EDL, and SOL muscles. (G) Weight of GAS, TA, EDL, and SOL muscles. (H) Muscles‐to‐body weight ratios of GAS, TA, EDL and SOL muscles. (I–K) Representative H&E staining (I), mean myofiber CSA (J) and the percentage of CSA distribution (K) of TA muscles. (L‐N) Representative H&E staining (L), mean myofiber CSA (M) and the percentage of CSA distribution (N) of SOL muscles. Age and body weight‐matched male adult OCN^−^/^−^ mice were intramuscularly injected into hindlimbs with adenovirus (AAV9‐Empty or AAV9‐Gprc6a) at 100 µL/mice. After 5 weeks, mice were immobilized for seven days and received rOCN administration or control daily. The rOCN was injected intraperitoneally at 30 ng/g body weight daily. Samples were collected at day seven post‐IMM. Representative images (scale bar = 50 µm) captured at 400× magnification. n = 6 for each group unless otherwise specified, data points show individual mice. Data are represented as mean ± SEM and were analyzed by two‐way ANOVA with Tukey's post‐hoc test. ^*^ *p* <0.05.^**^ *p* <0.01. The ^*^ in Figure D, K, N mean significant difference between Empty‐IMM‐rOCN vs AAV‐Gprc6a‐IMM‐rOCN.

Gprc6a is widely accepted as OCN's putative receptor in muscle, we next investigated whether OCN protects against muscle atrophy through Gprc6a. From three screened siRNAs, siRNA‐3 was chosen for its high knockdown efficiency (Figure S7A), and transfection of si‐Gprc6a‐3 abolished the rOCN‐mediated protective effect of against myotube atrophy (Figure S7E,F). AAV9 vectors encoding si‐Gprc6a‐3 (AAV‐Gprc6a) or negative control (Empty) were intramuscularly injected into bilateral hindlimbs of OCN^−/−^ mice. The specificity of this construct was confirmed in mouse skeletal muscle, where it knocked down the Gprc6a (Figure 6A) without affecting the expression of top‐predicted off‐target genes (Figure S7B). Unsurprisingly, AAV‐Gprc6a did not affect body weight (Figure S7G) or the exercise capacity (Figure S7H,I), but abolished the beneficial effects of rOCN on exercise (Figure S7J,K). Then we generated IMM model, and found that, although AAV‐Gprc6a did not affect body weight loss, it abolished rOCN‐mediated protective effect against IMM‐induced body weight loss (Figure 6B,C). In addition, rOCN failed to enhance food intake (Figure 6D,E), or alleviate muscle weight loss (Figure 6F,G) and muscle‐to‐body weight ratio (Figure 6H) in AAV‐Gprc6a mice. Moreover, rOCN treatment significantly increased mean CSA of TA (Figure 6I,J) and SOL (Figure 6L,M), and led to a rightward (larger) shift of CSA distribution in both TA (Figure 6K) and SOL (Figure 6N) in Empty mice, whereas these effects were absent in AAV‐Gprc6a mice. These data suggest that Gprc6a is the essential receptor of OCN in alleviating IMM‐induced muscle atrophy.

### 2.6 Fndc5 is a Potential Downstream Mediator of OCN in Muscle Atrophy

**FIGURE 7:**
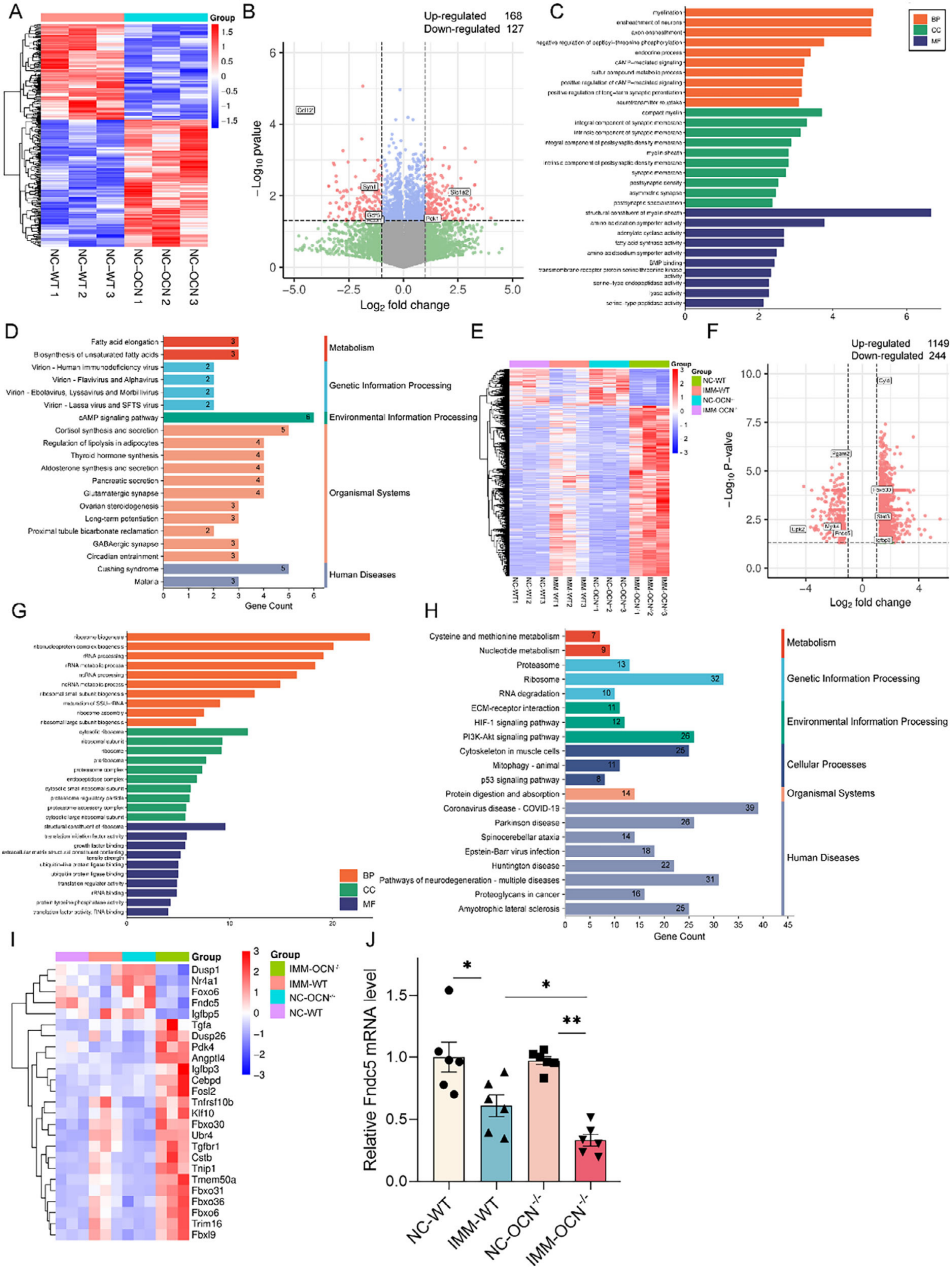
Fndc5 is a potential mediator of OCN deficiency‐induced muscle atrophy. (A‐D) Heatmap (A), Volcano (B), GO terms (C) and KEGG pathways (D) from NC‐WT vs NC‐OCN^−/−^. (E) Heatmap of OCN‐DEGs. (F‐H) Volcano (F), GO (G) and KEGG pathways (H) of OCN‐DEGs (represented by IMM‐WT vs IMM‐OCN^−/−^). (I) Heatmap of OCN‐DEGs involved muscle synthesis and degradation. (J) Relative Fndc5 levels (n = 6). TA muscles from NC‐WT, IMM‐WT, NC‐OCN^−/−^ and IMM‐OCN^−/−^ were used for transcriptome. n = 3 for each group unless otherwise specified, data points show individual mice. Data are represented as mean ± SEM and were analyzed by unpaired Two‐tailed Student's t test and two‐way ANOVA with Tukey's post hoc test. ^*^ *p* <0.05.^**^ *p* <0.01.

To comprehensively investigate the role of OCN deficiency in muscle homeostasis and elucidate how OCN deficiency exacerbates IMM‐induced muscle atrophy, we performed transcriptomic sequencing on TA muscles from NC‐WT, IMM‐WT, NC‐OCN^−/−^, IMM‐OCN^−/−^ mice. Between NC‐WT vs. NC‐OCN^−/−^, a total of 295 DEGs were identified (Figure 7A,B). GO and KEGG enrichment analyses revealed that OCN deficiency primarily altered genes associated with synaptic and myelin components (postsynaptic density, synaptic membrane, compact myelin) and metabolic/endocrine pathways (fatty acid synthesis/elongation, cAMP signaling, and cortisol/thyroid hormone synthesis). Notably, molecular functions such as BMP binding and transmembrane receptor serine/threonine kinase activity were enriched, suggesting dysregulated BMP/TGF‐β signaling (Figure 7C,D). These findings establish a role for OCN in maintaining basal metabolic and neuromuscular homeostasis.

We next evaluated how disuse induces muscle atrophy. Transcriptomic analysis in WT mice (NC‐WT vs. IMM‐WT) revealed 819 DEGs (Figure S8A,B). GO enrichment highlighted receptor/ligand and hormone activities, ion channel functions, and glucocorticoid/corticosteroid responses, along with neuronal processes and mechanical stimulus detection (Figure S8C), while KEGG analysis revealed MAPK, TGF‐β, TNF, and Rap1 signaling, reflecting stress, inflammatory, and catabolic pathways (Figure S8D). These results highlight the importance of steroid hormone responsiveness, altered ion channel activity, and stress/inflammation‐related pathways in IMM induces muscle atrophy.

We further explored the mechanism by which OCN deficiency exacerbates IMM‐induced muscle atrophy. OCN deficiency may amplify transcriptional responses to IMM by either amplifying the fold changes of those DEGs identified in WT mice and/or inducing novel DEGs. We thus identified those DEGs whose expression is modulated by OCN deficiency (OCN‐DEGs) in IMM‐induced muscle atrophy. A total of 1393 OCN‐DEGs were identified (Figure 7E,F). GO enrichment involved ribosomal constituents, translation initiation/regulation, rRNA binding, and ubiquitin/ubiquitin‐like ligase binding, indicating altered protein synthesis, translation, and proteostasis. Enrichment of growth factor binding and ECM structural components conferring tensile strength suggests potential remodeling of the muscle extracellular environment and impaired mechanotransduction. KEGG analysis highlighted ribosome, proteasome, ECM‐receptor interaction, cytoskeleton, mitophagy, HIF‐1, PI3K‐Akt, and p53 signaling pathways, reflecting disrupted protein homeostasis, mitochondrial quality control, and ECM integrity, along with activation of stress and catabolic signaling (Figure 7G,H). Among the 1393 OCN‐DEGs, we prioritized genes directly associated with protein synthesis and degradation (Figure 7I). Fndc5/Irisin is one of the OCN‐DEGs (Figure 7I,J) reported to ameliorate sarcopenia and metabolic dysfunction. It has also been reported to modulate PI3K‐Akt, HIF‐1, and ECM remodeling. Moreover, siRNA targeting Fndc5/Irisin (si‐Fndc5‐2) abolished the protective effect of rOCN against myotube atrophy (Figure S7C–F). Collectively, Fndc5/Irisin acts as a potential downstream effector of OCN in muscle atrophy.

### 2.7 Muscle Fndc5 Knockdown Abolishes OCN‐Mediated Protection Against Muscle Atrophy

**FIGURE 8:**
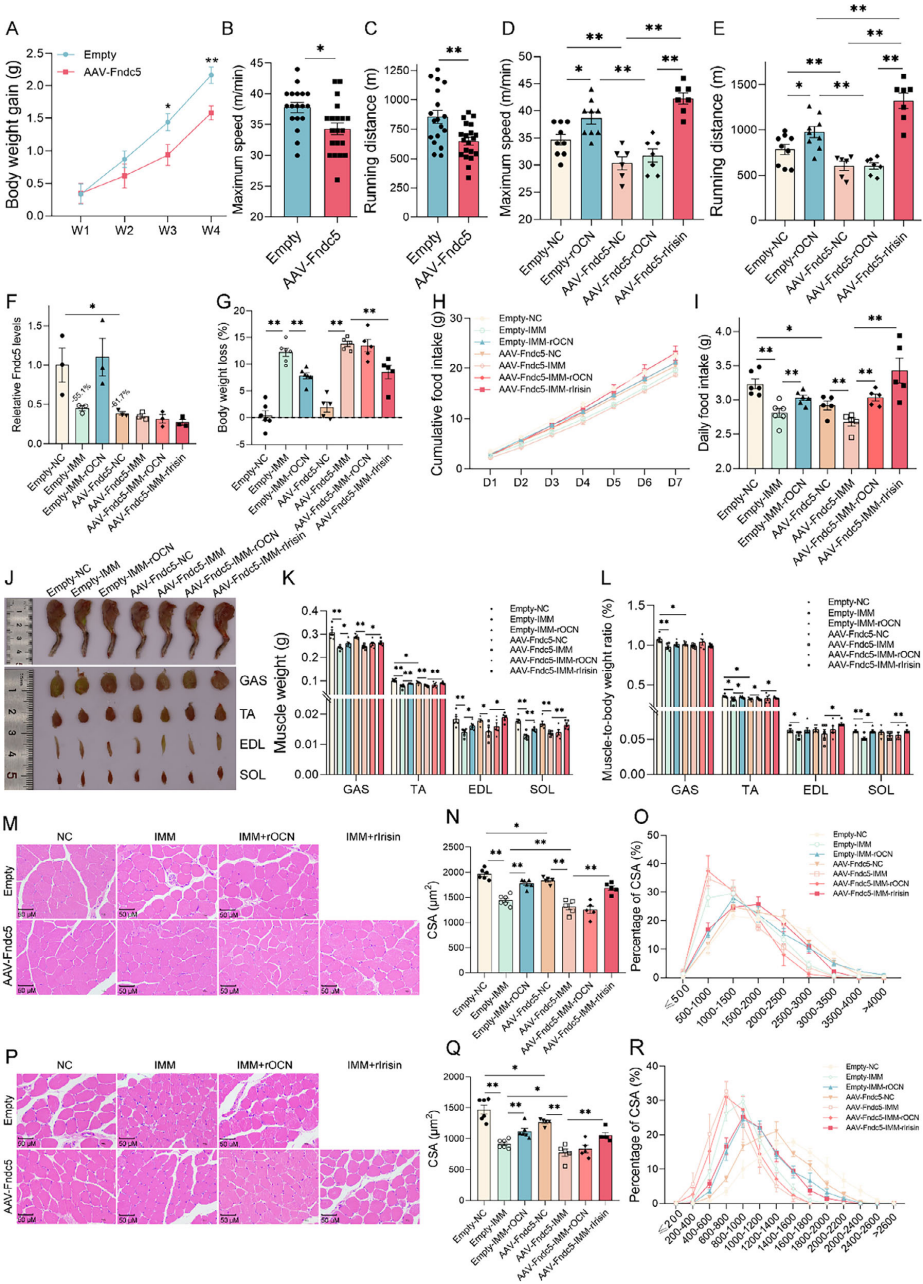
Muscle Fndc5 knockdown abolishes the beneficial effect of OCN in muscle atrophy. (A) Body weight gain (n = 18 for Empty group, n = 20 for AAV‐Fndc5 group). (B,C) Maximum speed (B) and running distance (C) (n = 18 for Empty group, n = 20 for AAV‐Fndc5 group). (D,E) Maximum speed (D) and running distance (E) receiving rOCN or rIrisin administration (n = 9 for Empty‐NC and Empty‐rOCN group, n = 6 for AAV‐Fndc5‐NC group, n = 7 for AAV‐Fndc5‐rOCN and AAV‐Fndc5‐rIrisin groups) (F) Relative Fndc5 mRNA levels in muscle, Gapdh was used as reference gene (n = 3 for each group). (G) Body weight loss. (H,I) Quantification of cumulative food intake (H) and daily food intake (I). (J) Representative images of hindlimbs and GAS, TA, EDL, and SOL muscles. (K) Weight of GAS, TA, EDL, and SOL muscles. (L) Muscle‐to‐body weight ratios of GAS, TA, EDL and SOL muscles. (M‐O) Representative H&E staining (M), mean myofiber CSA (N) and the percentage of CSA distribution (O) of TA muscles. (P–R) Representative H&E staining (P), mean myofiber CSA (Q) and the percentage of CSA distribution (R) of SOL muscles. Age and body weight‐matched male adult OCN^−/−^ mice were intramuscularly injected once into hindlimbs with adenovirus (AAV9‐Empty or AAV9‐Fndc5) at 100 µL/mice. After 4 weeks, mice were first acclimated to the treadmill for 3 days. On day 4, the effect of Fndc5 knockdown on exercise performance was evaluated. On day 6, the impact of rOCN or rIrisin on exercise capacity was examined. After 5 weeks, body weight‐matched mice were underwent seven‐day IMM and received daily treatments of either rOCN, rIrisin, or control. The rOCN was injected intraperitoneally at 30 ng/g body weight daily. The rIrisin was injected intraperitoneally at 0.5 µg/mouse/week daily. Thus, seven groups of mice (Empty‐NC, Empty‐IMM, Empty‐IMM‐rOCN, AAV‐Fndc5‐NC, AAV‐Fndc5‐IMM, AAV‐Fndc5‐IMM‐rOCN, AAV‐Fndc5‐IMM‐rIrisin) were studied here. Representative images (scale bar = 50 µm) captured at 400× magnification. n = 6 for Empty‐NC, Empty‐IMM and Empty‐IMM‐rOCN groups, n = 5 for AAV‐Fndc5‐NC, AAV‐Fndc5‐IMM, AAV‐Fndc5‐IMM‐rOCN and AAV‐Fndc5‐IMM‐rIrisin groups, unless otherwise specified. Data points show individual mice. Data are represented as mean ± SEM and analyzed by unpaired Two‐tailed Student's t tests or two‐way ANOVA with Tukey's post‐hoc test. ^*^ *p* <0.05.^**^ *p* <0.01.

We next examined whether the reduction of Fndc5/Irisin phenocopies IMM‐induced atrophy and, more critically, whether it functions as the downstream effector of OCN‐mediated protection. Remarkably, AAV9 vectors encoding si‐Fndc5‐2 (AAV‐Fndc5) suppressed body weight gain (Figure 8A; Figure S9A), exercise performance (Figure 8B,C), and abolished rOCN‐mediated beneficial effects in exercise (Figure 8D,E), alongside a 61.7% reduction of muscular Fndc5 (Figure ) without altering predicted off‐target genes (Figure S7D). Notably, rOCN reversed IMM‐induced Fndc5 decrease (Figure 8F) and body weight loss (Figure 8G) in Empty mice, confirming a functional targeting relationship. Besides, AAV‐Fndc5 also recapitulated key features of muscle atrophy under physiological condition, including reduced food intake (Figure 8H,I; Figure S9B), and decreased muscle weight (Figure 8J,K), muscle‐to‐body weight ratios (Figure 8L), myofiber CSA and led leftward (smaller) shift of CSA distribution in both TA (Figure 8M–O; Figure S9C) and SOL muscles (Figure 8P–R; Figure S9F), highlight its critical role in muscle mass and function. More importantly, AAV‐Fndc5 abolished the protective effects of rOCN against IMM‐induced loss of body weight (Figure 8G), muscle weight (Figure 8J,K), muscle‐to‐body weight ratios (Figure 8L), and myofiber CSA of TA (Figure 8M–O; Figure S9D,E) and SOL muscles (Figure 8P–R; Figure S9G,H). In contrast, the exogenous rIrisin restored exercise capacity (Figure 8D,E) and ameliorated IMM‐induced muscle atrophy, as evidenced by increased muscle weight (Figure 8J,K), muscle‐to‐body weight ratios (Figure 8L), myofiber CSA and leading a rightward (larger) shift of CSA distribution in both TA (Figure 8M–O; Figure S9E) and SOL muscles (Figure 8P–R; Figure S9H), collectively establishing Fndc5/Irisin as an essential mediator of OCN. Interestingly, rOCN‐induced orexigenic effect was not abolished by AAV‐Fndc5 (Figure 8H,I; Figure S9B). Collectively, these findings establish Fndc5/Irisin as essential for OCN‐mediated protection against muscle atrophy.

Based on our findings that OCN deficiency exacerbated muscle atrophy via further reducing Fndc5 levels, we hypothesized that Fndc5/Irisin might alleviate IMM‐induced muscle atrophy in a dose‐dependent manner. Notably, rIrisin administrations at doses of 0.5 µg/mouse/week (0.5 rIrisin) and 5 µg/mouse/week (5 rIrisin) elevated serum irisin levels in a dose‐dependent manner (Figure S10A). rlrisin administration exhibited dose‐dependent efficacy in alleviating body weight loss, with 5 rIrisin eliciting superior protection compared to 0.5 rIrisin (Figure S10B,C). Similarly, both doses increased cumulative food intake and daily food intake, with 5 rIrisin showing stronger promotion (Figure S10D,E). Besides, 5 rIrisin exhibited superior protection in muscle weight and muscle‐to‐body weight ratio of GAS compared with 0.5 rIrisin (Figure S10F–H). Moreover, rIrisin increased mean CSA and led rightward (larger) shift in both TA (Figure S10I–K) and SOL (Figure S10L–N) in dose‐dependent manner. Notably, a significant difference was observed in mean CSA of TA (Figure S10I,J) and SOL (Figure S10L,M), and rightward (larger) shift of SOL (Figure S10N) between 0.5 rIrisin and 5 rIrisin, suggesting that Fndc5/Irisin ameliorates IMM‐induced muscle atrophy in dose‐dependent manner.

### 2.8 OCN/FNDC5 Axis is Functionally Conserved in Pig

Having observed protection of OCN/Fndc5 axis against muscle atrophy in mice, we next sought to determine whether this mechanism is evolutionarily conserved. We analyzed the sequence conservation of OCN (*BGLAP1/2*) and *FNDC5*, and found that both *BGLAP1/2* (Figure S11A) and *FNDC5* (Figure S11B) are highly conserved among mice, human and pig. Given the economic significance, we focused on pigs and analyzed muscle transcriptome data from pigs (GSE157044) to investigate whether *FNDC5* is involved in muscle atrophy/myogenesis (OCN was excluded from muscle‐specific analysis due to its absence in muscle tissue). Porcine muscle transcriptome data across developmental stages showed a significant upregulation of *FNDC5* expression during the first 30 days after birth (Figure S11C), suggesting a potential role of *FNDC5* in pig. Based on these findings, we attempted to investigate the functional role of OCN/FNDC5 axis in pig.

**FIGURE 9:**
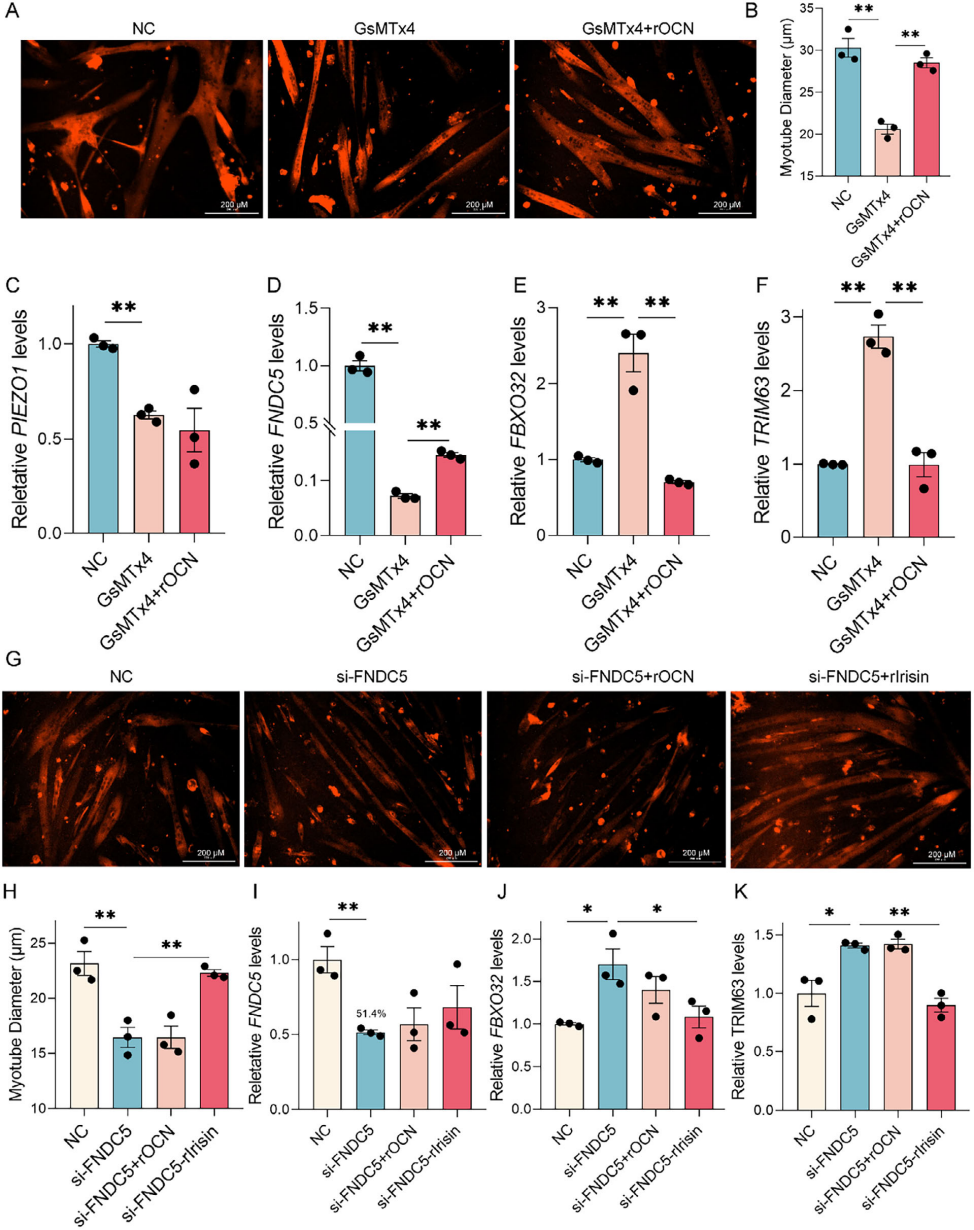
OCN ameliorates PIEZO1 inactivation‐induced muscle atrophy in FNDC5‐dependent manner in pig. (A,B) Representative immunofluorescence staining of MYHC (A) and quantification of myotube diameters (B) of GsMTx4, GsMTx4+rOCN or control myotubes. (C–F) Relative mRNA levels of porcine PIEZO1 (C), FNDC5 (D), FBXO32 (E) and TRIM63 (F), GAPDH was used as reference gene. (G,H) Representative immunofluorescence staining of MYHC (G) and quantification of myotube diameters (H) in si‐FNDC5, si‐FNDC5+rOCN, si‐FNDC5+rIrisin or control myotubes. (I–K) Relative mRNA levels of porcine FNDC5 (I), FBXO32 (J) and TRIM63 (K), GAPDH was used as reference gene. The porcine primary myotubes (differentiated for three days) were used. The GsMTx4 was used at a concentration of 5 µm. The rOCN war used at 10 ng/mL. The rIrisin were used at 0.5 µg/mL. The si‐FNDC5 was transfected at 20 nm. Representative images (scale bar = 200 µm) captured at 10× magnification. n = 3 for each group, data points show individual mice. Data are represented as mean ± SEM and were analyzed by one‐way ANOVA with Dunnett's multiple comparisons test or Tukey's post‐hoc test. ^*^ *p* <0.05.^**^ *p* <0.01.

We first employed porcine primary myotubes (MSCs purity at 93.45% ± 1.73%, Figure S11D) to assess whether disuse (PIEZO1 inactivation) leads to muscle atrophy in pig. The GsMTx4 (PIEZO1 inhibitor) administration significantly reduced myotube diameter, which was markedly rescued by rOCN (Figure 9A,B). Besides, GsMTx4 administration led to significant downregulations of both *PIEZO1* (Figure 9C) and *FNDC5* (Figure 9D), and upregulations of the muscle atrophy markers *FBXO32* (Figure 9E) and *TRIM63* (Figure 9F). Notably, rOCN restored *FNDC5* expression (Figure 9D) while attenuated *FBXO32* (Figure 9E) and *TRIM63* (Figure 9F) levels, without affecting *PIEZO1* level (Figure 9C), suggesting that OCN acts downstream of Piezo1 to mitigate muscle atrophy in pigs. To further assess whether OCN exerts its protective effects through *FNDC5*, we inhibited *FNDC5* by siRNA (si‐FNDC5). si‐FNDC5 significantly decreased myotube diameter (Figure 9G,H) and *FNDC5* levels (∼50%) (Figure 9I), but increased *FBXO32* and *TRIM63* levels (Figure 9J,K), highlighting the value of *FNDC5* in the muscle mass of pig. Notably, rOCN failed to ameliorate si‐FNDC5‐induced myotube atrophy as rIrisin did (Figure 9G,H,J,K), demonstrating that FNDC5 is required for OCN‐mediated protection. Collectively, these findings demonstrate that the OCN/FNDC5 axis is functionally conserved in pigs.

## 3 Discussion

Accumulating evidence demonstrates that bone functions as an endocrine organ, secreting osteokines such as OCN, to regulate muscle metabolism and exercise adaptation. However, whether OCN participates in disuse‐induced muscle atrophy, and through what mechanism, remains controversial. Here, we identify a mechanosensitive Piezo1/OCN/Irisin endocrine axis that links skeletal mechanotransduction to muscle mass homeostasis during disuse. We show that mechanical unloading is associated with suppressed bone Piezo1 expression, reducing circulating unOCN levels and exacerbating muscle atrophy. Exogenous OCN attenuated muscle atrophy and accelerated recovery via its muscle receptor Gprc6a. Critically, Fndc5/Irisin serves as a key downstream effector of OCN, evidenced by rIrisin attenuate muscle atrophy in OCN^−/−^ mice, whereas muscle Fndc5/Irisin knockdown abolishes the benefic effects of OCN against muscle atrophy. These findings provide clear evidence that OCN protects against muscle atrophy.

Disuse‐induced muscle atrophy leads to loss of muscle strength, impaired functional capacity, increased risk of metabolic disorders, and susceptibility to injuries and comorbidities [30]. Previous research mainly focuses on myokines‐mediated muscle homeostasis [31], but how the systemic crosstalk, particularly bone‐muscle interactions, affects muscle atrophy remains largely understudied. OCN, a bone‐derived hormone that functions in undercarboxylated form, enhances muscle function and metabolism [9] and stimulates in vitro myogenesis [10]. OCN garnered both interest and controversy in muscle proteostasis. Some studies report no observable effect of OCN on muscle mass in young healthy rodents [13, 14, 15], and minimal effect in the unilateral hindlimb immobilization mouse model [16]. In our study, exogenous OCN administration or genetic OCN deficiency modulated exercise capacity without altering muscle mass at physiological conditions, consistent with the notion that OCN is not a primary driver of basal muscle hypertrophy. In contrast, in the IMM‐induced muscle atrophy model, exogenous OCN significantly attenuated muscle loss, whereas OCN deficiency exacerbated muscle wasting, highlighting the protection of OCN in pathological stress.

We further demonstrated that OCN is involved in muscle recovery. rOCN treatment significantly accelerated recovery of atrophic muscle in OCN^−/−^ mice within seven days, whereas a prolonged intervention (14 days) was required to induce an observable difference in WT mice. This temporal difference between OCN^−/−^ and WT mice may help explain previous controversies regarding OCN's role in muscle. The minimal effect observed in unilateral immobilization models [16] may be influenced by compensatory loading‐induced OCN secretion from the non‐immobilized limb, which potentially masks the efficacy of exogenous supplementation. In contrast, the bilateral immobilization model completely abolishes hindlimb loading, creating a context where OCN deficiency becomes critical and exogenous supplementation becomes therapeutically effective.

To clarify how disuse decreases OCN levels, we analyzed skeletal transcriptomes from hindlimb unloading (HLU) mice (GSE235942), which revealed disuse‐induced DEGs were enriched in ion channel and PI3K/Akt pathway. Consistent with this, previous studies reported that mechanical unloading impairs bone remodeling via inactivating Piezo1‐Ca^2^^+^ cascades [32], and PI3K/Akt signaling is downstream effector of Piezo1 in bone‐related disease [33]. Supporting this, Piezo1 knockdown in osteoblasts reduced Bglap (OCN) expression [34], while Yoda1‐induced Piezo1 activation restored serum OCN in HLU mice [35], suggesting Piezo1 may serve as an upstream regulator of OCN in disuse models. To test whether skeletal Piezo1 serves as an upstream regulator of OCN and protects against muscle atrophy in the disuse model, we knockdown skeletal Piezo1 and administrated mice with Piezo1 agonist Yoda1. Knockdown of skeletal Piezo1 abolished Yoda1‐mediated protections on IMM‐induced muscle atrophy, demonstrating the value role of skeletal Piezo1. While Yoda1 administration cannot protected muscle atrophy in OCN^−/−^ mice, highlighting that the benefits of skeletal Piezo1 are, at least partially, dependent on OCN. Contrary to previous work that Yoda1‐mediated Piezo1 activation alleviates muscle atrophy through maintaining basal muscular Ca^2+^ in vivo and in vitro [1], our data support a distinct endocrine route in which Piezo1 activation in bone engages OCN to influence muscle mass. It is possible that the relative contribution of bone‐mediated versus muscle‐intrinsic Piezo1 mechanisms depends on unloading duration, tissue responsiveness, and experimental context.

We next sought to elucidate how the skeletal muscle senses OCN signal. OCN functions through receptors Gprc6a [9, 11], Gpr37 [29], and Gpr158 [36], of which Gprc6a is the receptor in skeletal muscle [9, 11]. To determine whether OCN alleviates muscle atrophy through Gprc6a signaling, we used AAV9 to knockdown muscular Gprc6a in mice. Consistent with previous studies, Gprc6a knockdown abolished the beneficial effects of OCN on muscle atrophy, indicating that Gprc6a is required for OCN‐mediated anti‐atrophic signaling in skeletal muscle.

Exercise‐inducible OCN and Fndc5/Irisin [9, 19] reveals striking functional parallels in multi‐tissue homeostasis. However, direct evidence linking skeletal OCN to muscular Fndc5/Irisin remains limited. Our transcriptomic data suggested that OCN deficiency potentially aggravated muscle atrophy by reducing Fndc5/Irisin levels, thereby establishing a potential link between OCN and Fndc5/Irisin. OCN deficiency did not affect muscle mass, while muscular Fndc5/Irisin knockdown recapitulated key features of IMM‐induced muscle atrophy and abolished the protective effects of exogenous OCN. Importantly, exogenous rIrisin rescued muscle mass and function in AAV‐Fndc5 mice, this unidirectional dependency strongly supports that Fndc5/Irisin acts as a critical downstream effector in OCN‐mediated muscular protection. Together, these findings suggest that Fndc5/Irisin is an essential downstream mediator of OCN in protecting against muscle atrophy, and that exogenous Irisin can restore muscle protection in the absence of OCN.

The orexigenic effect of OCN raised the question of whether its protection against disuse‐induced muscle atrophy is mediated primarily through increased food intake or via a direct action on skeletal muscle. Our data support a direct anti‐atrophic mechanism independent of appetite stimulation. Using a pair‐feeding approach in OCN‐deficient mice, we found that even when food intake was strictly controlled to match that of vehicle‐treated controls, exogenous OCN significantly ameliorated muscle atrophy, as evidenced by preserved muscle mass and myofiber CSA. While ad libitum feeding further enhanced these protective effects, suggesting a synergistic contribution from increased caloric intake, the significant protection observed under pair‐fed conditions demonstrates that OCN's anti‐atrophic action is at least partly independent of its orexigenic function. This conclusion is further supported by genetic evidence from muscle‐specific Fndc5‐knockdown mice, in which the anti‐atrophic effects of OCN were completely abolished while its orexigenic effect was preserved. This phenotypic uncoupling indicates that appetite stimulation alone is insufficient to confer muscle protection and that OCN's anti‐atrophic action requires an intact Fndc5/Irisin pathway in skeletal muscle. Collectively, these physiological (pair‐feeding) and genetic (Fndc5 knockdown) approaches suggest that OCN protects against IMM‐induced muscle atrophy predominantly through a direct, Fndc5‐dependent pathway in skeletal muscle.

We further evaluated the functionally conserved role of *PIEZO1/OCN/FNDC5* across species. In modern intensive pig production systems, high stocking density may compromise muscle development due to restricted physical activity. Specifically, we show that rOCN ameliorates *PIEZO1* inactivation‐induced myotube atrophy, while *FNDC5* knockdown abolishes this protective effect. These findings confirm the PIEZO1/OCN/Irisin axis is functionally conserved in porcine myotubes, providing a potential strategy to mitigate muscle growth impairment under intensive farming conditions.

Despite these promising findings, this study has several limitations that should be acknowledged. First, although AAV‐mediated tissue‐specific knockdown allowed us to decrease gene expression level in targeted tissues and produced functional effects, this approach has limitations compared with genetic conditional knockout models. Viral infection may not be completely restricted to a single cell type within the targeted tissue, and the knockdown efficiency is incomplete, which may underestimate the full contribution of the target gene. Second, although our results support a signaling pathway linking Piezo1 to OCN and downstream Fndc5/Irisin, the detailed molecular mechanisms remain unclear. Specifically, how skeletal Piezo1 regulates Bglap expression, and how OCN signaling controls Fndc5 expression and muscle protein homeostasis, require further investigation. Third, while OCN's direct anti‐atrophic effect can be separated from its appetite‐stimulating effect, as shown by pair‐feeding and Fndc5 knockdown experiments, it remains unclear whether the additional benefit observed under ad libitum feeding is simply due to increased caloric intake or involves other OCN‐mediated systemic mechanisms remains unclear. Finally, although we observed functional conservation of this axis in porcine primary myotubes, in vivo studies in large animals and further investigation in human disuse conditions will be necessary to determine its clinical relevance.

In summary, we identify a mechanosensitive Piezo1/osteocalcin/Irisin axis that links bone mechanotransduction to muscle homeostasis under disuse conditions. Mechanical unloading suppresses bone Piezo1 expression and circulating unOCN, leading to impaired Fndc5/Irisin signaling and muscle atrophy. Restoring this axis via Piezo1 activation or exogenous OCN/Irisin attenuated muscle atrophy, whereas muscle‐specific knockdown of Gprc6a or Fndc5 abolished OCN‐mediated protective effects. These findings establish OCN as a mechanoresponsive hormone and highlight the Piezo1/OCN/Irisin axis as a promising therapeutic target for disuse‐induced muscle atrophy.

## 4 Methods

### 4.1 Ethical Approval

Animal handling and treatment were approved by the Committee of Experimental Animal Management at Northwest A&F University (approval ID: XN2024‐0305).

### 4.2 Animal Studies

All mice were housed in the animal facility at Northwest A&F University under standard conditions with free access to food and water. The light was on from 8 am to 8 pm, with the temperature kept at 21 °C–24 °C and humidity at 40%–70%. Mice were euthanized by CO_2_ inhalation following AVMA and institutional guidelines. Blood samples were immediately collected via cardiac puncture. Tissue samples were immediately formalin‐fixed for subsequent histological evaluation or snap‐frozen and stored at −80 °C.

### 4.3 Generation of OCN (Bglap/Bglap2) Deletion Mice Using CRISPR/Cas9

The OCN knockout mice are designed and generated by Cyagen biosciences (Suzhou) inc (Suzhou, China). Briefly, the gRNA (gRNA1 target sequence: GCATGGTGTTGCTACCCTCCA GG; gRNA2 target sequence: CTCTGCGTCCCCTGTTCCACTGG) to the mouse Bglap1/2 gene, and Cas9 mRNA were co‐injected into fertilized mouse eggs to generate targeted knockout offspring. F0 founder animals were identified by PCR followed by sequence analysis, which were bred to wildtype mice to test germline transmission and F1 animal generation. Inter‐cross heterozygous mice to generate homozygous mice.

### 4.4 Generation of Bilateral Hindlimbs IMM‐Induced Muscle Atrophy

Age and body weight‐matched littermate C57BL/6J mice were used to generate IMM‐induced muscle atrophy. Before IMM, mice were anesthetized with isoflurane, and sufficient anaesthetization was checked via the footpad pinch test. Bilateral hindlimbs were gently positioned in a natural weight‐bearing posture to prevent joint hyperextension. The hindlimbs were protected with cotton gauze, and immobilized using a cast. Cast integrity, limb swelling, and general health (activity, responsiveness, appearance) were monitored daily during IMM.

### 4.5 Food Intake Measurement

Mice were individually housed, and move freely in the cage to obtain food and water. Food consumption was recorded daily. Cumulative food intake was calculated by summing the daily food consumption. Daily food intake was determined by dividing the cumulative food intake by immobilization days. Measurements were performed at 16:00 daily.

### 4.6 Administration of Recombinant OCN (rOCN), Recombinant Irisin (rIrisin), and Yoda1

Following immobilization procedure on Day 0, rOCN (RPA471Mu01, USCN Life Science, Wuhan, China) was administered via daily intraperitoneal injection (IP) at 30 ng/g body weight, rIrisin (HY‐P70665, MCE, USA) was administered via daily IP at a dose of 0.5 or 5 µg/mouse/week. 0.5% (w/v) BSA was used as a control. The Yoda1 (SML1558, Sigma, USA) was administered via daily IP at a dose of 0.2 mg/kg body weight.

### 4.7 Pair‐Feeding Experiment

Male OCN^−/−^ mice at 8 weeks of age were individually housed and acclimated to feeding conditions for 5 days, during which baseline body weight and food intake were recorded. Mice were then randomly assigned to one of three experimental groups based on body weight and baseline food intake: (1) vehicle with ad libitum feeding (NC‐Ad libitum), (2) rOCN with pair‐feeding to NC‐Ad libitum (rOCN‐Pair fed), and (3) rOCN with ad libitum feeding (rOCN‐Ad libitum). All mice were subsequently subjected to IMM to induce muscle atrophy. Throughout the 7‐day IMM period, mice in the rOCN‐Pair fed group received a daily amount of food equal to the average intake of the NC‐Ad libitum group on the preceding day, ensuring matched caloric intake between these groups. Mice in the rOCN‐Ad libitum group had unrestricted access to food. All mice had free access to water throughout the experiment.

### 4.8 Serum OCN and Irisin Measurement

Blood collected from mice were centrifuged at 1500×*g* for 15 min at 4 °C, and serum was collected. Undercarboxylated OCN level in the serum was quantified using a commercial ELISA kit (ml037462, Shanghai Enzyme‐linked Biotechnology Co., Ltd, Shanghai, China). Irisin level in the serum were quantified using commercial ELISA kit (F10940, Xitang Biotechnology Co., Ltd, Shanghai, China).

### 4.9 AAV‐Mediated Knockdown of Gprc6a, Fndc5, and Piezo1

To knockdown Gprc6a and Fndc5 in skeletal muscle, three independent siRNA sequences were designed and screened for each target gene in cultured C2C12 myotubes. The sequence showing the highest knockdown efficiency was selected and subsequently cloned into an AAV9 vector (Hanbio, Shanghai, China). We then transduced AAV9 shRNA against Gprc6a and Fndc5 into age and body weight‐matched adult littermate OCN^−/−^ mice. Scramble shRNA was used as the negative control (Empty). AAV was diluted in saline to 1 × 10^12^ vector genomes/mL, and 100 µL/mouse was injected into bilateral hindlimbs. 4 weeks after AAV injection, mice received further treatment to evaluated the effect of Gprc6a/Fndc5 in muscle atrophy. To assess potential off‐target effects, the selected shRNA sequences (si‐Gprc6a‐3 and si‐Fndc5‐2) were analyzed using BLAST against the mouse transcriptome. The top two predicted off‐target genes for each shRNA (Atp8b2 and Csnk2a2ip for Gprc6a, and Casp12 and Trpm3 for Fndc5) were subsequently evaluated by quantitative PCR in skeletal muscle samples from AAV‐treated mice to exclude nonspecific silencing.

To knockdown skeletal Piezo1, AAV9 vectors encoding short hairpin RNA targeting mouse Piezo1 (AAV‐Piezo1) or a scrambled negative control sequence (Empty) were constructed (Hanbio, Shanghai, China). The shRNA sequence against Piezo1 was designed based on a previously validated sequence [37]. AAV‐Piezo1 or the corresponding empty vector was injected via the intramedullary route into the bilateral hindlimbs tibias of wild type male mice at the age of 8 weeks. All the target sequences are listed in Table S1.

### 4.10 Histological Analysis

The histological analysis was performed as previously described [38]. Briefly, the TA and SOL muscles were fixed with 4% paraformaldehyde more than 24 h and subjected to hematoxylin‐eosin (H&E) staining. The CSA was calculated using ImageJ for ∼5 fields of each view and 5 views for each.

The tibiofibula bones were used for osteoblasts staining. Following fixation, tissues were decalcified in 10% EDTA solution (YE0105, Bomeibio, China). Decalcified tissues underwent automated dehydration through a graded series (75% ethanol for 2 h, 85% for 1 h, 95% for 1 h, 100% ethanol, renewal every 20 min × 4 times, clearing reagents for 25 min followed 30 min, paraffin for 30 min, followed 1 h × 2 times) before embedding, sectioning and deparaffinizing. Staining was performed with hematoxylin for 5–10 min until wash under running water, differentiated in acid alcohol for 3s, wash under running water, incubation with weak alkaline solution, then counterstained with alcoholic eosin for 3 min. Sections were dehydrated through graded alcohols, cleared in xylene, and mounted with neutral balsam. Images were acquired using a 3DHISTECH scanner (Pannoramic 250, Hungary), manual counting was used to count the number of osteoblasts in three 200X images.

TRAP was used for osteoclasts analysis. The deparaffinized tibiofibula bones sections were circled with Pap Pen, the sections were incubated with distilled water at 37 °C for 2 h in a humidified chamber. Following water removal, freshly prepared and filtered TRAP staining solution was applied and incubated at 37 °C for 30–60 min. After staining, sections were rinsed with water, counterstained with hematoxylin for 1–2 min, differentiated in 1% acid alcohol, and incubation in ammonia water. Finally, the sections were dehydrated through graded ethanol, cleared in xylene, and mounted with neutral balsam. Images were acquired using a 3DHISTECH scanner (Pannoramic 250, Hungary), and manual counting was used to count the number of osteoclasts in three 200X images.

### 4.11 Cell Culture and Treatment

The primary porcine muscle stem cells (MSCs) were isolated from male DLY piglets (within 3‐day‐old) and cultured in RPMI 1640 medium (11875093, Gibco) containing 20% fetal bovine serum (FBS) (FBS‐AUS050, SERANA), 1% penicillin‐streptomycin (P1400, Solarbio), 1% chicken embryo extract (abs80002, Absin), and 1% FGFβ (GMP‐C046, Novoprotein) as our previously described [39]. The C2C12 myoblast cells were provided by the Chinese Academy of Sciences (Shanghai, China) and cultured in DMEM (SH30243.01, HyClone, USA) containing 10% FBS. When the cell density reached 90%, the cells were cultured with differentiation medium DMEM containing 2% horse serum (S9050, Solarbio, China). For transfection, the siRNAs targeting mice Gprc6a, Fndc5 or porcine FNDC5 were mixed with lipofectamine 2000 Reagent (11668019, Invitrogen, USA) and transfected into cells as the manufacture protocol described. The siRNA sequences are listed in Table S1. The GsMTx4 (HY‐P1410, MCE, USA) was used to mimic disuse‐induced Piezo1 inactivation at a concentration of 5 µm. For recombinant protein administration, the rOCN and rIrisin were treated at 10 ng/mL and 0.5 µg/mL, respectively. All cells were cultured at 37 °C in an atmosphere with 5% CO_2_.

### 4.12 RNA Isolation and Reverse Transcription‐Quantitative Polymerase Chain Reaction (RT‐qPCR)

The total RNA from cells, muscles and tibiofibula bone were isolated by RNA Extraction Kit (AG21024, Accurate Biology, China) according to the instructions. RT‐qPCR were performed as previously described [40]. The primer sequences of genes detected in this study are listed in Table S2.

### 4.13 Immunofluorescence Staining

The Immunofluorescence staining of MyHC and Pax7 were performed according to our previously described methods [41]. Images were obtained from a Cell Imaging Microplate Detection System (Bio Tek, Cytation5) and quantified by Image J. The information of antibodies is listed in Table S3.

### 4.14 Bioinformatics Analysis

Total RNA was isolated from of TA muscles using standard Trizol protocol, then the transcriptome sequencing was performed on DNBSEQ‐T7 platform by BGI.tec (BGI lnc., Shenzhen, China). Genes with a log2 fold change ≥1.25 and *p* ≤0.05 were considered differentially expressed.

### 4.15 Exercise Test

For exercise studies, all mice were trained to run on a treadmill for 3 days (10 min/day, with increasing speed from 10 to 20 m/min at an acceleration rate of 2 m/min, and an electric shock at 0.1 mA to trigger running). For the maximum speed test, mice were acclimated to the treadmill for 3 min, followed with increasing running speed by 2 m/min until exhaustion. For the maximum running distance test, mice were acclimated to the treadmill for 3 min. The speed was then gradually increased from 10 to 20 m/min, followed by a 20 min running at 20 m/min. Mice ran either until exhaustion or further increased running speed by 2 m/min every 5 min. Mouse was removed from the electric grid if a mouse falls off the grid 15 times within 30 s.

### 4.16 Indirect Calorimetry Experiment

The indirect calorimetry between OCN^−/−^ and littermate WT mice was determined in an indirect calorimetric system from Columbus Instruments International Corporation (Oxymax). Following a 24‐h acclimatization, oxygen consumption, carbon dioxide production, energy expenditure, and respiratory exchange ratio (RER) were continuously monitored for full 24 h.

### 4.17 Statistical Analysis

All data were assessed for normality and homogeneity of variance using the Shapiro‐Wilk test and F‐test, respectively, to validate the assumptions for the parametric tests used. No outliers were identified or removed in this study. Data were presented as mean ± standard error of the mean (SEM). The sample size (n) for each experimental group is provided in the corresponding figure legends and represents biological replicates from independent experiments. Statistical comparisons between two groups were performed using an unpaired two‐tailed Student's t‐test. For comparisons involving more than two groups, one‐way ANOVA followed by Dunnett's post‐hoc test (when multiple groups were compared to a single control) or Tukey's post‐hoc test (for all pairwise comparisons within an experiment) was applied. When two independent factors were involved, data were analyzed by two‐way ANOVA followed by Tukey's multiple comparisons test. Statistical analysis was performed using GraphPad Prism version 8.0.2 for Windows (GraphPad Software, USA, www.graphpad.com). *p* < 0.05 was considered significant. ^*^ *p* < 0.05; ^**^ *p* < 0.01.

## Author Contributions

Z.L.W and X.E.S conceived the study. Z.L.W designed the study. Z.L.W., X.S., X.Y.J and H.C performed the experiments. Z.L.W analyzed the data and wrote the manuscript. X.E.S and J.J.J revised the manuscript. All authors reviewed and approved the final manuscript.

## Conflicts of Interest

The authors declare no conflicts of interest.

## Supporting information




**Supporting File**: advs75355‐sup‐0001‐SuppMat.docx.

## Data Availability

The raw sequencing data generated in this study have been deposited in the NCBI Sequence Read Archive (SRA) under BioProject accession number PRJNA1322151. The data will be publicly available on September 1, 2027 at: https://www.ncbi.nlm.nih.gov/sra/PRJNA1322151. Other data and materials are provided within the manuscript and supplementary materials, or available upon reasonable request to the corresponding author (Xin'e Shi, xineshi@nwafu.edu.cn).
