# Supplementary material for: Mechanosensitive Piezo1/Osteocalcin/Irisin Axis Protects Against Disuse‐Induced Muscle Atrophy
Source: Adv Sci (Weinh). 2026 Apr 17;13(39):e75355. doi: 10.1002/advs.75355 (PMC13335622; doi:10.1002/advs.75355)
Supplement: Supplementary file 1 — Supporting File: advs75355‐sup‐0001‐SuppMat.docx. [file ADVS-13-e75355-s001.docx]

**Supplementary information**

**Supplementary Figures and Figure legend**

**Fig. S1 Bilateral hindlimb immobilization (IMM) induce muscle atrophy**


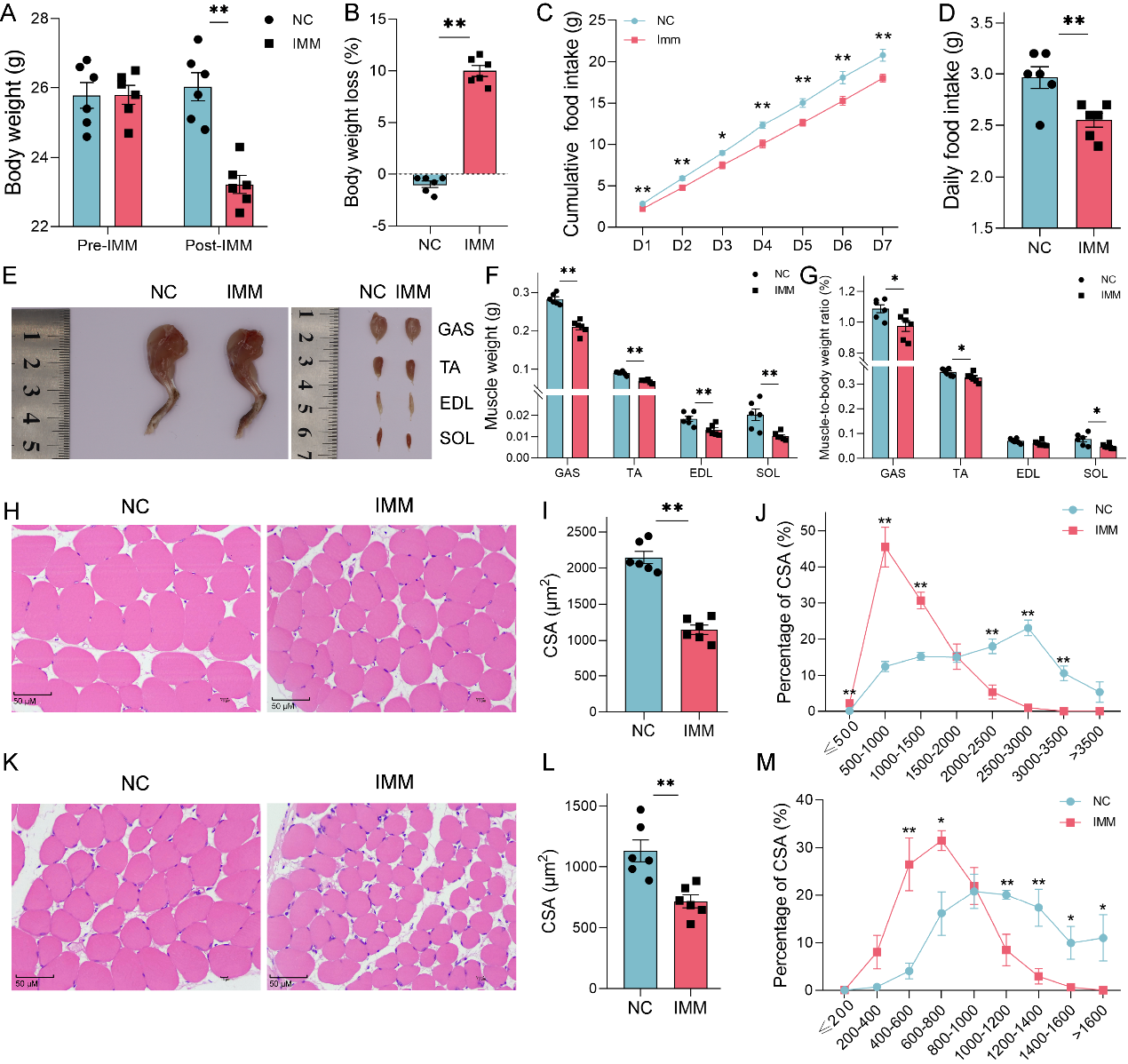


(A and B) Body weight change (A) and body weight loss (B). (C and D) Quantification of cumulative food intake (C) and daily food intake (D). (E) Representative images of hindlimbs and GAS, TA, EDL, and SOL muscles. (F) Weight of GAS, TA, EDL, and SOL muscles. (G) Muscle-to-body weight ratios of GAS, TA, EDL and SOL from bilateral hindlimbs. (H-J) Representative H&E staining (H), mean myofiber cross-sectional area (CSA) (I) and the percentage of CSA distribution (J) of TA muscles. (K-M) Representative H&E staining (K), mean myofiber CSA (L) and the percentage of CSA distribution (M) of SOL muscles.

Age and body weight-matched WT male mice (8 weeks of age) were used to generate bilateral hindlimbs IMM-induced muscle atrophy model. Samples were collected at day 7 post-IMM. Representative images (scale bar = 50 μm) captured at 400× magnification. n=6 for each group unless otherwise specified, data points show individual mice. Data are represented as mean ± SEM and were analyzed by unpaired Two-tailed Student's *t* tests. * *p* < 0.05.** *p* < 0.01.

**Fig. S2 Exogenous OCN promotes muscle function without affecting muscle mass under physiological conditions**


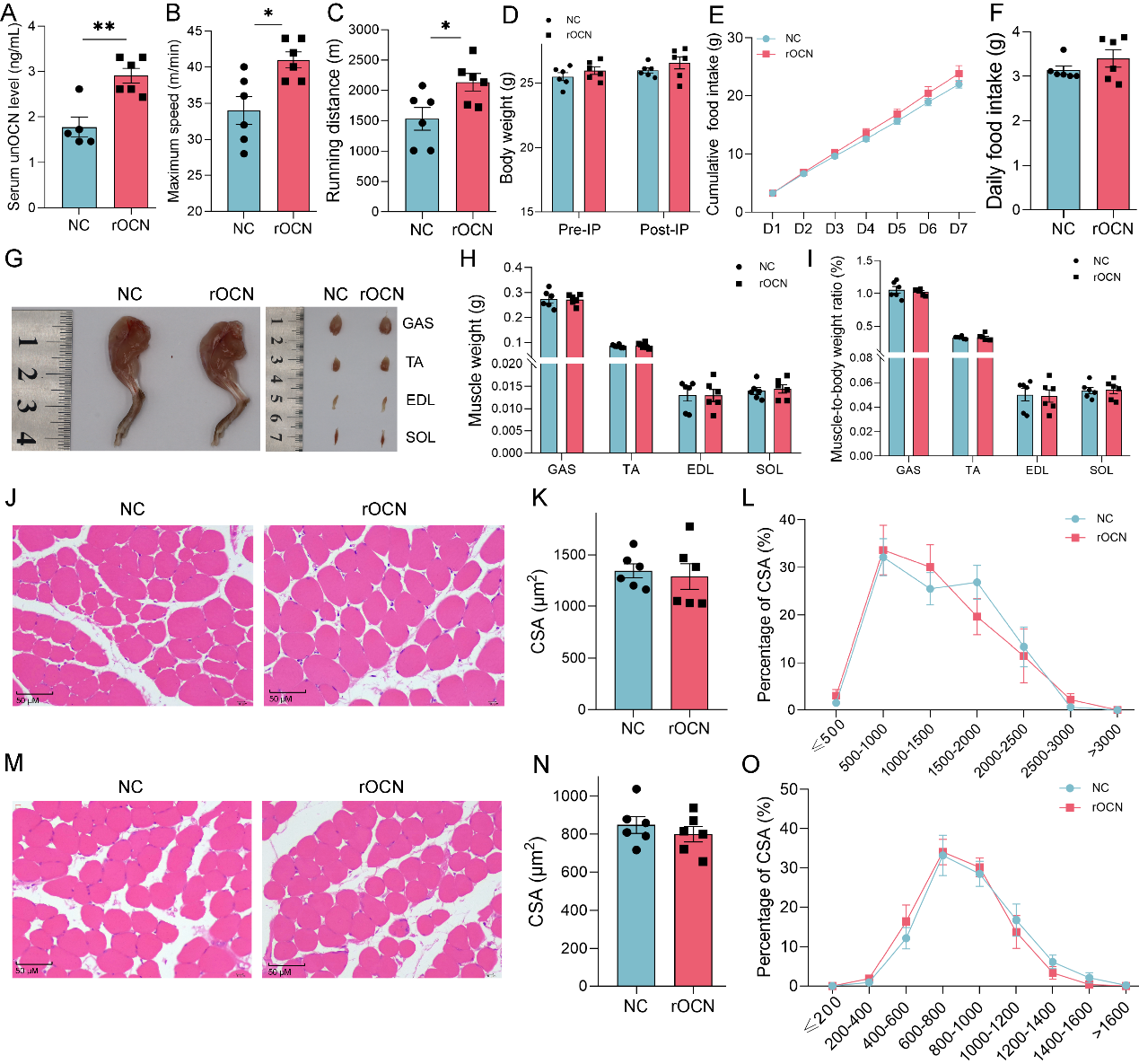


(A) Serum unOCN levels from mice receiving daily intraperitoneal injection of rOCN in non-atrophic mice (n = 5 for NC group, n = 6 for rOCN group) (B and C) Maximum speed (B) and running distance (C) of mice receiving a single dose of rOCN administration. (D) Body weight changes. (E and F) Quantification of cumulative food intake (E) and daily food intake (F). (G) Representative images of hindlimbs and GAS, TA, EDL, and SOL muscles. (H) Weight of GAS, TA, EDL, and SOL muscles. (I) Muscle-to-body weight ratios of GAS, TA, EDL and SOL muscles. (J-L) Representative H&E staining (J), mean myofiber CSA (K) and the percentage of CSA distribution (L) of TA muscles. (M-O) Representative H&E staining (M), mean myofiber CSA (N) and percentage of CSA distribution (O) of SOL muscles.

Age and body weight-matched WT male adult mice were daily IP of rOCN at 30 ng/g body weight at physiological state (non-atrophy). Samples were collected at day seven post-IMM. Representative images (scale bar = 50 μm) captured at 400× magnification. n=6 for each group unless otherwise specified, data points show individual mice. Data are represented as mean ± SEM and were analyzed by unpaired Two-tailed Student's *t* tests. * *p* < 0.05.** *p* < 0.01.

**Fig. S3 OCN deficiency impairs muscle function without altering muscle mass at 3 months of age**


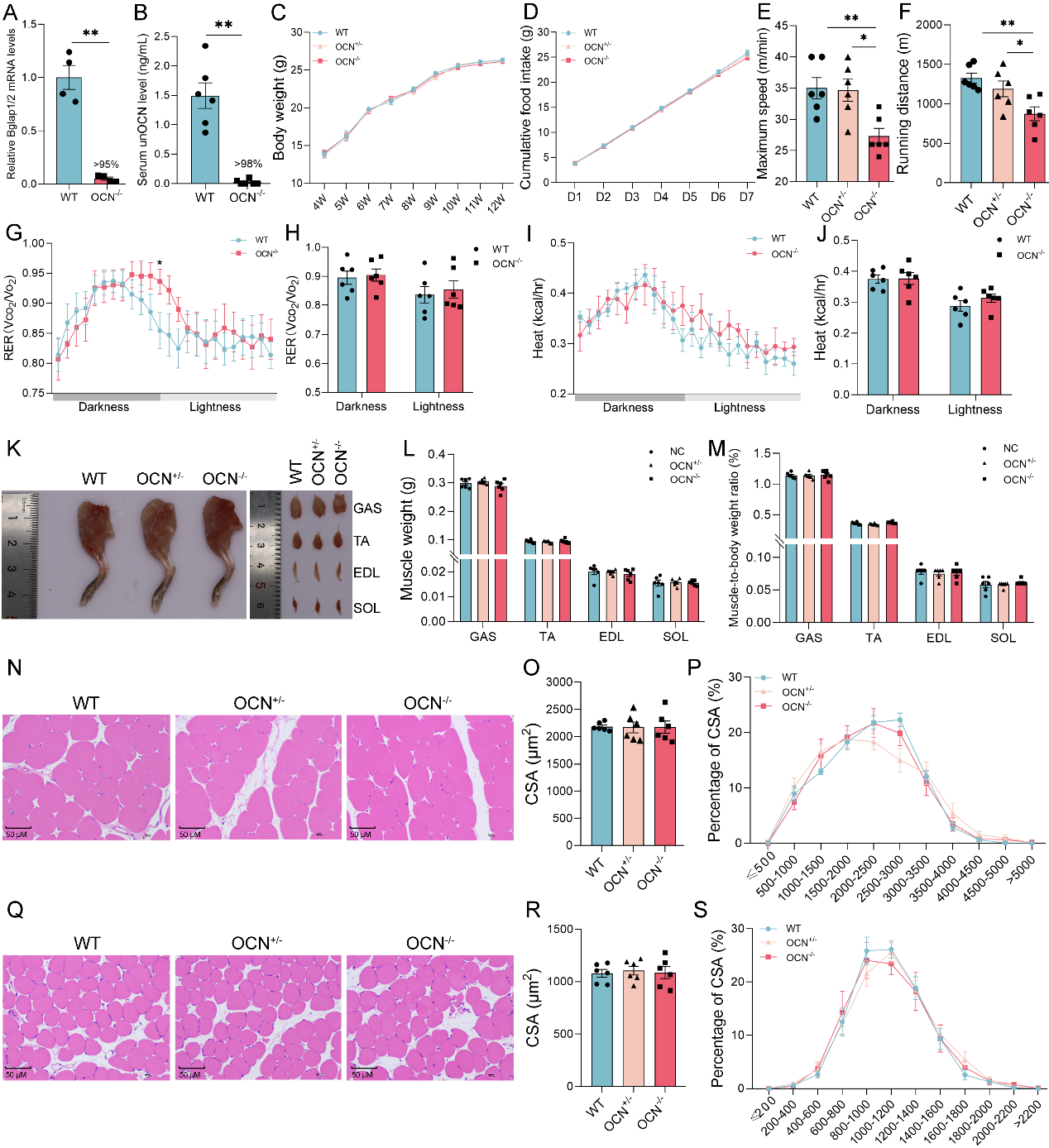


(A, B) Bone Bglap1/2 mRNA level (n = 4) (A) and serum unOCN between WT mice and their littermate OCN^-/-^ mice at age of 12 week. (C) Body weight from 4W to 12W (D) Quantification of cumulative food intake was evaluated at age 11w for seven days. (E and F) Maximum speed (E) and running distance (F) (G and H) Respiratory exchange ratio (RER) measurements (G) and statistical analysis (H) in WT and littermate OCN^-/-^ mice. (I and J) Heat production measurements (I) and statistical analysis (J) in WT and littermate OCN^-/-^ mice. (K) Representative images of hindlimbs and GAS, TA, EDL, and SOL muscles. (L) Weight of GAS, TA, EDL, and SOL muscles. (M) Muscle-to-body weight ratios of GAS, TA, EDL and SOL muscles. (N-P) Representative H&E staining (N), mean myofiber CSA (O) and the percentage of CSA distribution (P) of TA muscles. (Q-S) Representative H&E staining (Q), mean myofiber CSA (R) and the percentage of CSA distribution (S) of SOL muscles.

Age-matched male WT and their littermate OCN^+/-^ and OCN^-/-^ mice were used to evaluate the effect of OCN deficiency. Representative images (scale bar = 50 μm) captured at 400× magnification. n=6 for each group unless otherwise specified, data points show individual mice. Data are represented as mean ± SEM and were analyzed by Two-tailed Student's *t* tests (for two groups) or one-way ANOVA with Tukey's post hoc test (for three groups). * *p* < 0.05.** *p* < 0.01.

**Fig. S4 Exogenous OCN directly ameliorates IMM-induced muscle atrophy**


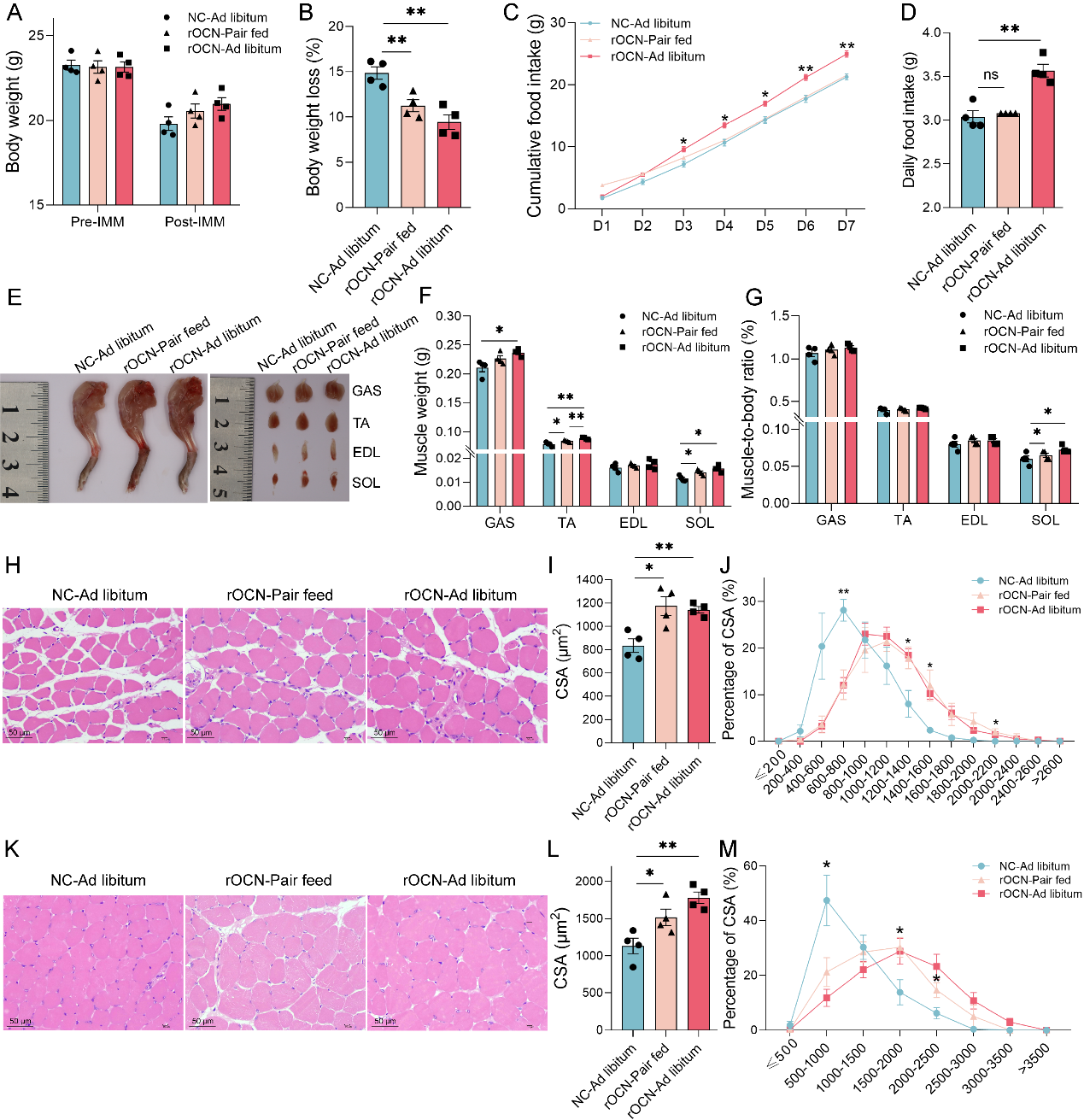


(A and B) Body weight changes (A) and body weight loss (B) of mice during IMM. (C and D) Quantification of cumulative food intake (C) and daily food intake (D). (E) Representative images of hindlimbs and GAS, TA, EDL, and SOL muscles. (F) Weight of GAS, TA, EDL, and SOL muscles. (G) Muscle-to-body weight ratios of GAS, TA, EDL and SOL muscles. (H-J) Representative H&E staining (H), mean myofiber CSA (I) and the percentage of CSA distribution (J) of TA muscles. (K-M) Representative H&E staining (K), mean myofiber CSA (L) and the percentage of CSA distribution (M) of SOL muscles.

8-weeks-old male OCN⁻^/^⁻ mice were acclimated for 5 days and randomized by body weight and baseline food intake into three groups: (1) vehicle with ad libitum feeding (NC-Ad libitum), (2) rOCN with pair-feeding to Vehicle Ad libitum (rOCN-Pair fed), and (3) rOCN with ad libitum feeding (rOCN-Ad libitum). All mice were subjected to IMM for 7 days under respective feeding plan. The rOCN was injected intraperitoneally at 30 ng/g body weight daily. Representative images (scale bar = 50 μm) captured at 400× magnification. n=4 for each group, data points show individual mice. Data are represented as mean ± SEM and were analyzed by one-way ANOVA followed by Tukey's post hoc test. * *p* < 0.05.** *p* < 0.01.

**Fig. S5 Exogenous OCN promotes the recovery of atrophied muscles in WT mice**


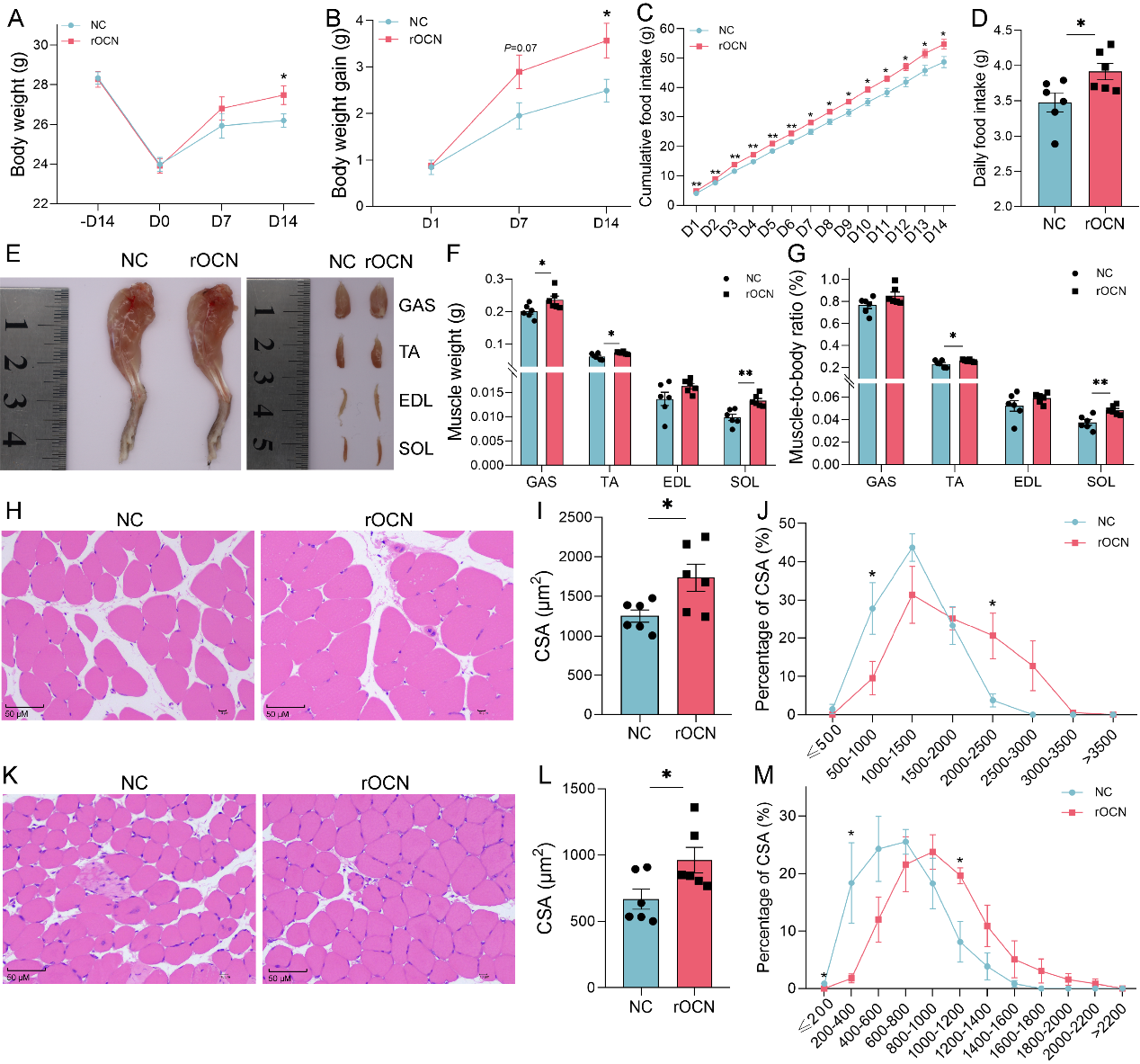


(A and B) Body weight changes of mice during 14-day IMM and subsequent 14-day recovery (A) and body weight gain from D0 to D14 (B). (C and D) Quantification of cumulative food intake (C) and daily food intake (D). (E) Representative images of hindlimbs and GAS, TA, EDL, and SOL muscles. (F) Weight of GAS, TA, EDL, and SOL muscles. (G) Muscle-to-body weight ratios of GAS, TA, EDL and SOL muscles. (H-J) Representative H&E staining (H), mean myofiber CSA (I) and the percentage of CSA distribution (J) of TA muscles. (K-M) Representative H&E staining (K), mean myofiber CSA (L) and the percentage of CSA distribution (M) of SOL muscles.

Age and body weight-matched male adult WT mice underwent 14 days of IMM to induce severe muscle atrophy, followed by removal of the IMM (Defined as Day 0, D0). Mice with similar body weight before and after IMM were randomly assigned to group receiving rOCN administration for 14 days. The rOCN was intraperitoneally injected at 30 ng/g body weight daily. Samples were collected at D14 post-IMM removal. Representative images (scale bar = 50 μm) captured at 400× magnification. n=6 for each group, data points show individual mice. Data are represented as mean ± SEM and were analyzed by unpaired Two-tailed Student's *t* tests. * *p* < 0.05.** *p* < 0.01.

**Fig. S6 Disuse induces bone transcriptome changes**


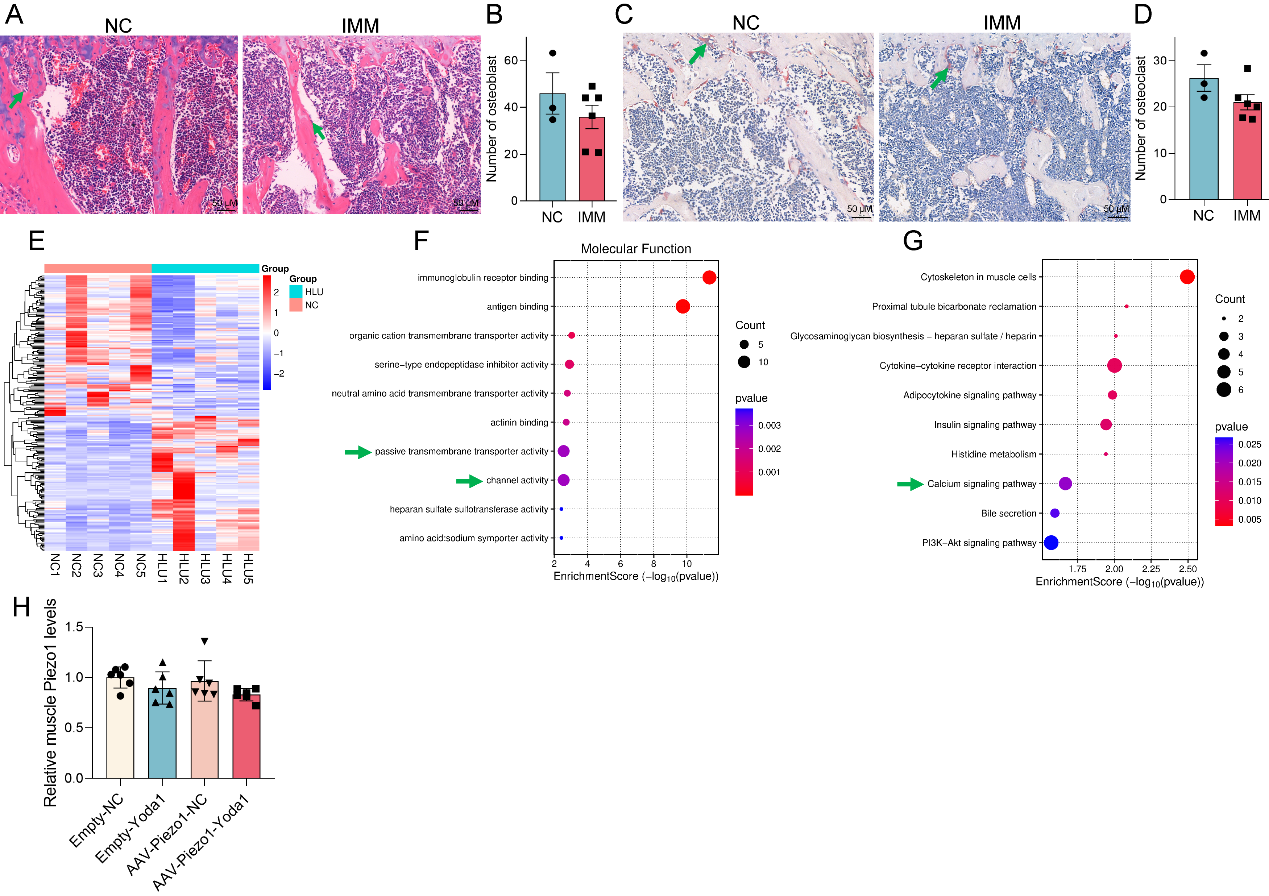


(A and B) Representative H&E staining (A) and quantification (B) of osteoblast number (Green arrows point to osteoblasts) (n = 3 for NC group, n = 6 for IMM group). (C and D) Representative TRAP staining (C) and quantification (D) of osteoclast number (Green arrows point to osteoclasts) (n = 3 for NC group, n = 6 for IMM group). (E-G) Bone transcriptomic heatmap (E) GO terms (F) and KEGG pathways (G) of GSE235942 (male, 6-month-old, n = 5 for each group). (H) Relative Piezo1 expression levels in muscle following Yoda1 administration during IMM of AAV-shPiezo1-treated mice (n = 6 for each group).

For Fig. A-D, Age and body weight-matched WT male mice (~8 weeks of age) underwent seven days of IMM to induce muscle atrophy, the tibiofibula bones were collected. Representative images (scale bar = 50 μm) captured at 400× magnification. For Fig. H, body weight-matched male WT mice at age of 8 week were used. AAV-shPiezo1 or the empty vector was injected via intramedullary route in both tibias. IMM-induced muscle atrophy model was generated at 5 weeks post-AAV-shPiezo1 injection. The Yoda1 was injected intraperitoneally at dose of 0.2 mg/kg body weight daily during IMM. TA muscles were collected at day seven post-IMM. Number of samples per group is specified in the corresponding figure legend, data points show individual mice. Data are represented as mean ± SEM and were analyzed by unpaired Two-tailed Student's *t* tests and two-way ANOVA followed by Sidak's multiple comparisons test for pre-selected comparisons. * *p* < 0.05.** *p* < 0.01.

**Fig. S7 Gprc6a and Fndc5 is necessary for OCN-mediated effects**


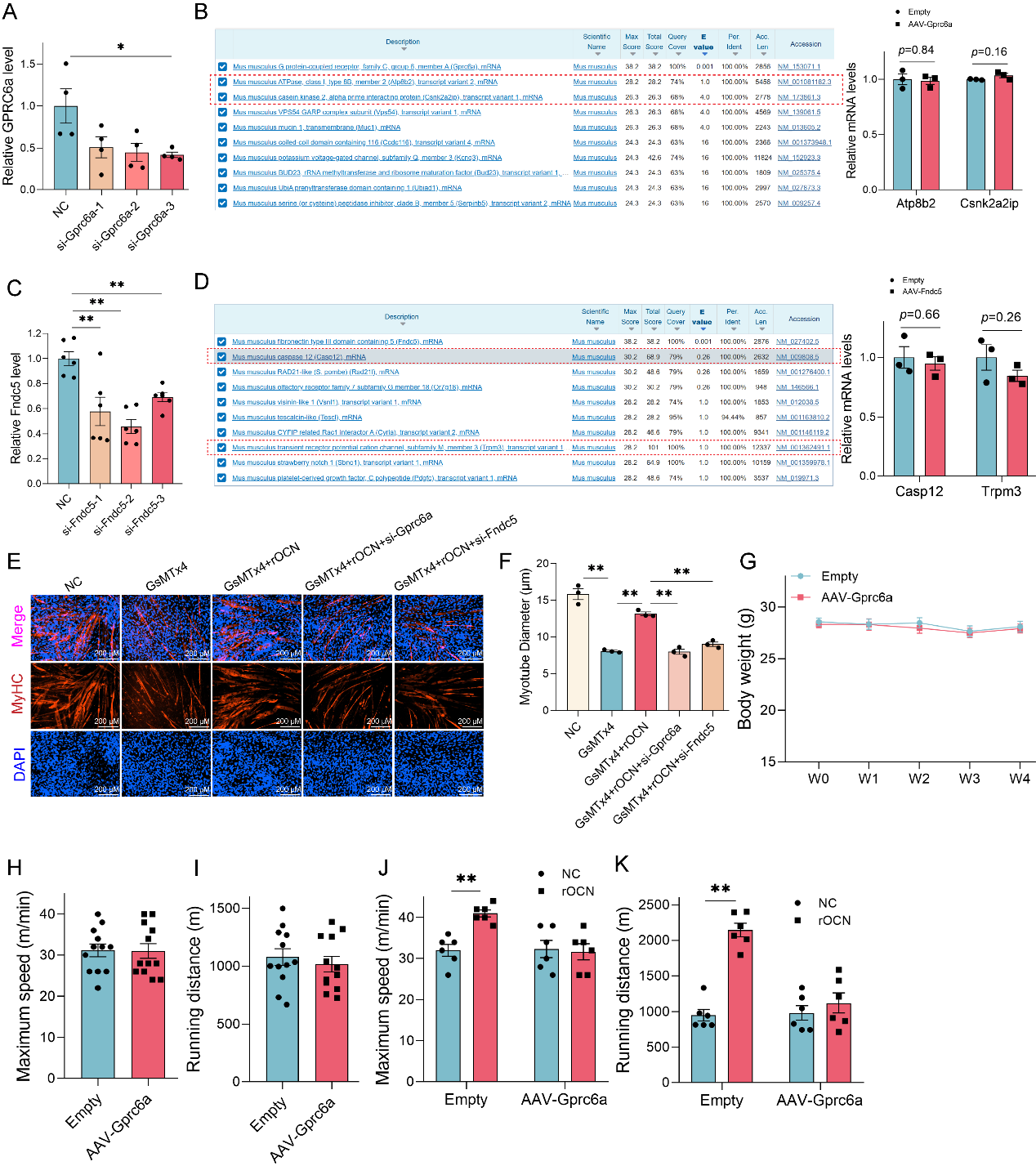


(A) Relative Gprc6a mRNA levels in C2C12 myotubes under siRNA treatments (n = 4 for each group). (B) Relative mRNA levels for BLAST-predicted off-target genes in AAV-Gprc6a-treated TA muscle (n = 3 for each group). (C) Relative Fndc5 mRNA levels in C2C12 myotubes under siRNA treatments (n = 6 for each group). (D) Relative mRNA levels for BLAST-predicted off-target genes in AAV-Fndc5-treated TA muscle (n = 3 for each group). (E and F) Representative immunofluorescence staining of MyHC (E) and quantification of myotube diameters (F) (n = 3 for each group). (G) Body weight changes of AAV-Gprc6a-treated mice. (H and I) Maximum speed (H) and running distance (I) of AAV-Gprc6a-treated mice. (J and K) Maximum speed (J) and running distance (K) of AAV-Gprc6a-treated mice receiving a single dose of rOCN administration (n = 12 for each group in Fig. G, H and I. n = 6 for each group in Fig. J and K).

Murine C2C12 myoblasts at day four post-differentiation were used to test the effects of Gprc6a and Fndc5 under GsMTx4 administration. All siRNAs were transfected with Lip2000 at 20 nM. The GsMTx4 was used at a concentration of 5 μM. The rOCN was used at 10 ng/mL. Age and body weight-matched male adult OCN^-/-^ mice were intramuscularly injected with adenovirus (AAV9-Empty or AAV9-Gprc6a) at 100 μL/mouse. Body weight was monitored weekly, and the effects of Gprc6a knockdown with or without rOCN on exercise capacity was test 4 weeks later. Representative images (scale bar = 200 μm) captured at 10× magnification. Number of samples per group is specified in the corresponding figure legend, data points show individual mice. Data are represented as mean ± SEM and were analyzed by unpaired Two-tailed Student's *t* tests or one-way ANOVA with Dunnett's multiple comparisons test. * *p* < 0.05.** *p* < 0.01.

**Fig. S8 Transcriptome signature of IMM-induced muscle atrophy in WT mice**


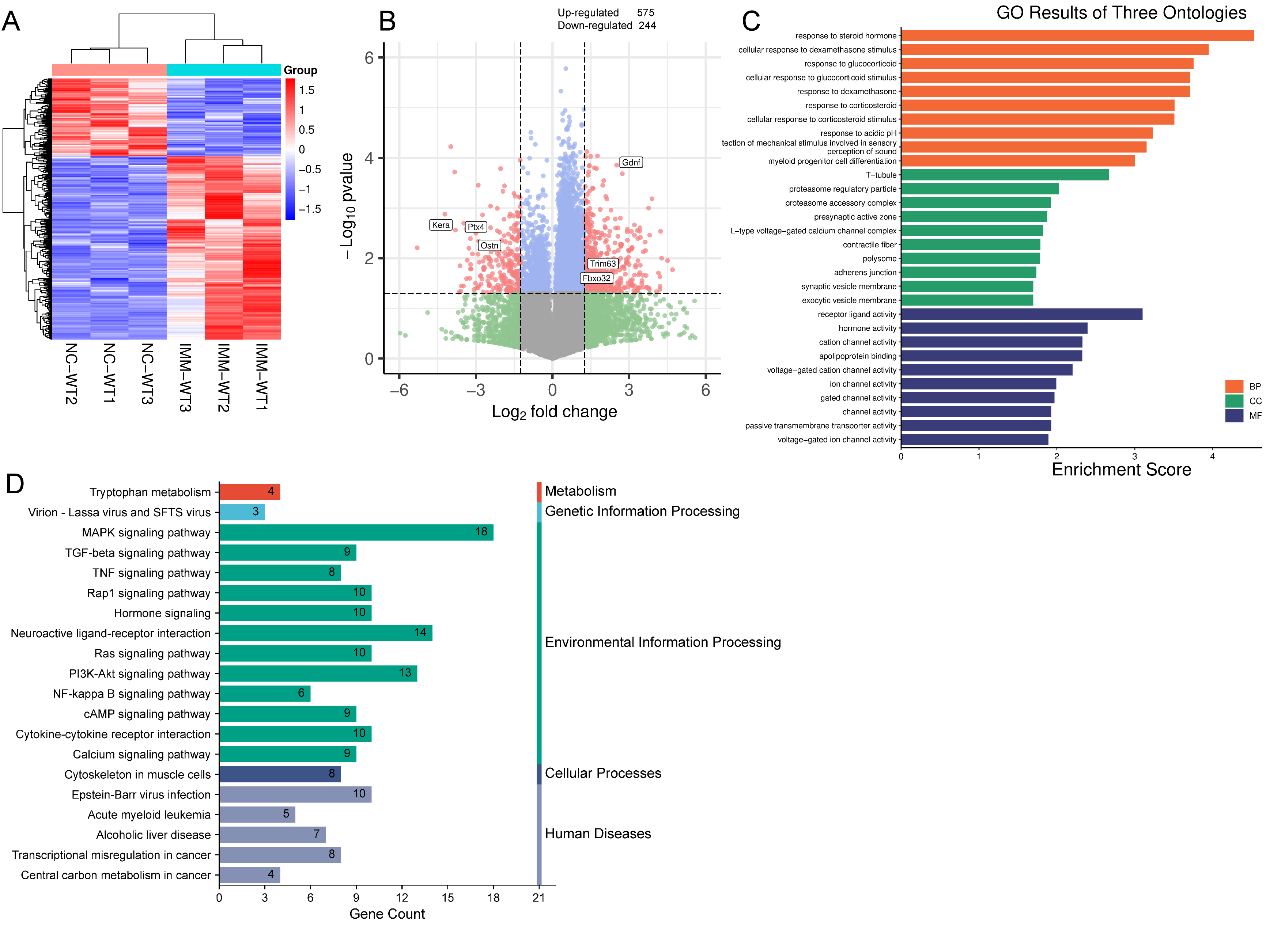


(A-E) Transcriptomic heatmap (A), Volcano (B), GO terms (C) and KEGG pathways (D) of TA from NC-WT vs IMM-WT.

Age and body weight-matched male WT adult mice were used. TA muscles were collected at day seven post-IMM. n = 3 for each group. Genes with a log2 fold change (Log2FC) ≥ 1.25 and P ≤ 0.05 were considered differentially expressed.

**Fig. S9 Muscle Fndc5 knockdown induced muscle atrophy and abolishes the beneficial effect of OCN against muscle atrophy**


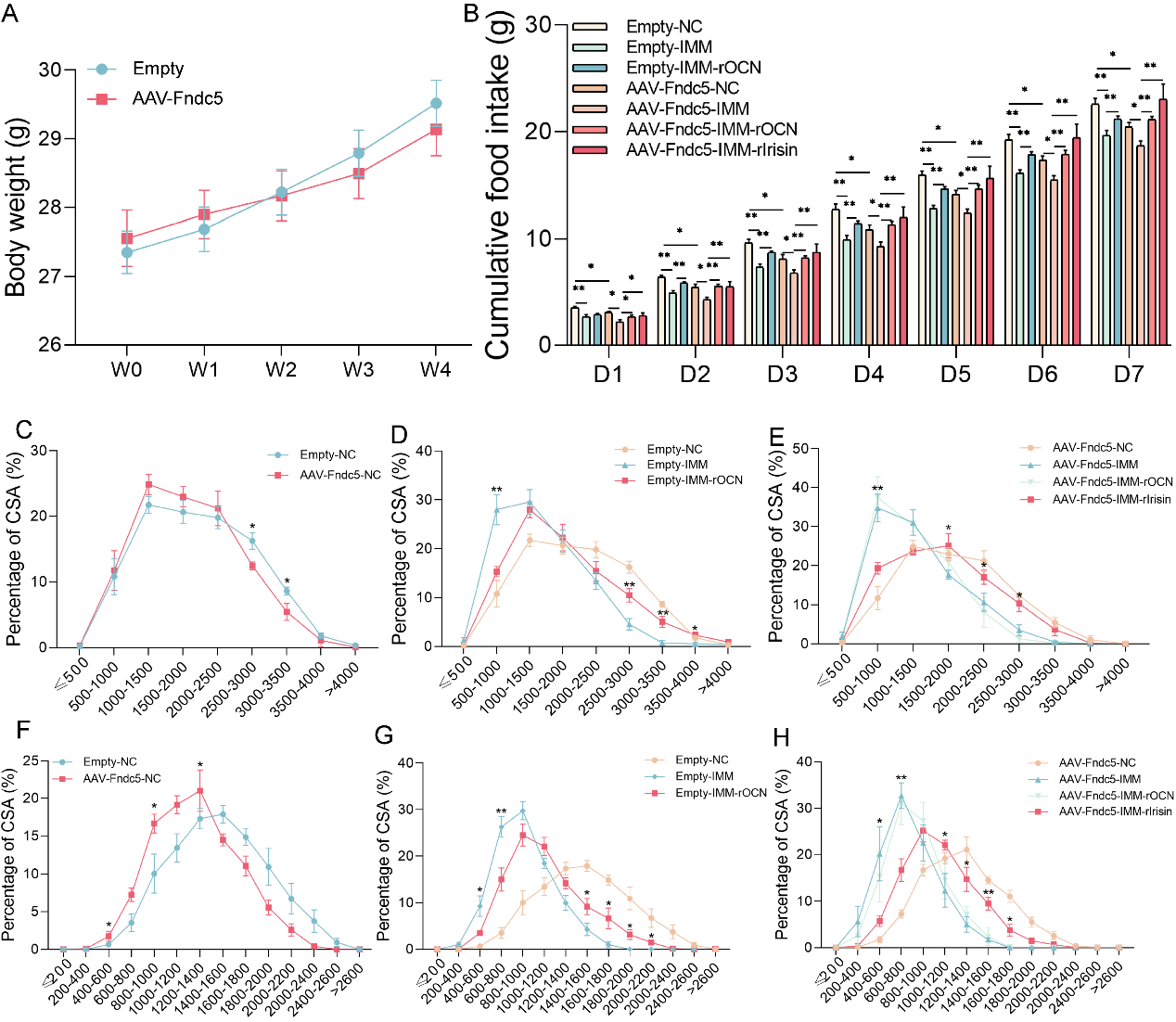


(A) Body weight changes post-AAV-Fndc5 injection (n = 18 for Empty group, n = 20 for AAV-Fndc5 group). (B) Quantification of cumulative food intake. (C-E) The percentage of CSA distribution in Empty-NC vs AAV-Fndc5-NC (C), Empty-NC vs Empty-IMM vs Empty-IMM-rOCN (D) and AAV-Fndc5-NC vs AAV-Fndc5-IMM vs AAV-Fndc5-IMM-rOCN vs AAV-Fndc5-IMM-rIrisin (E) of TA muscles. (F-H) The percentage of CSA distribution in Empty-NC vs AAV-Fndc5-NC (F), Empty-NC vs Empty-IMM vs Empty-IMM-rOCN (G) and AAV-Fndc5-NC vs AAV-Fndc5-IMM vs AAV-Fndc5-IMM-rOCN vs AAV-Fndc5-IMM-rIrisin (H) of SOL muscles.

Age and body weight-matched male adult OCN^-/-^ mice were intramuscularly injected once into hindlimbs with adenovirus (AAV-Empty or AAV-Fndc5) at 100 μL/mice. After 5 weeks of AAV-Fndc5 injection, body weight-matched mice were under IMM condition or basal for seven days. The rOCN was injected intraperitoneally at 30 ng/g body weight daily. The rIrisin was injected intraperitoneally at 0.5 μg/mouse/week daily. n = 6 for Empty-NC, Empty-IMM and Empty-IMM-rOCN groups, n = 5 for AAV-Fndc5-NC, AAV-Fndc5-IMM, AAV-Fndc5-IMM-rOCN and AAV-Fndc5-IMM-rIrisin groups, unless otherwise specified. Data are represented as mean ± SEM and were analyzed by unpaired Two-tailed Student's *t* tests or two-way ANOVA tests with Tukey’s post-hoc test. * *p* < 0.05.** *p* < 0.01.

**Fig. S10 rIrisin alleviates IMM-induced muscle atrophy in dose-dependent manner**


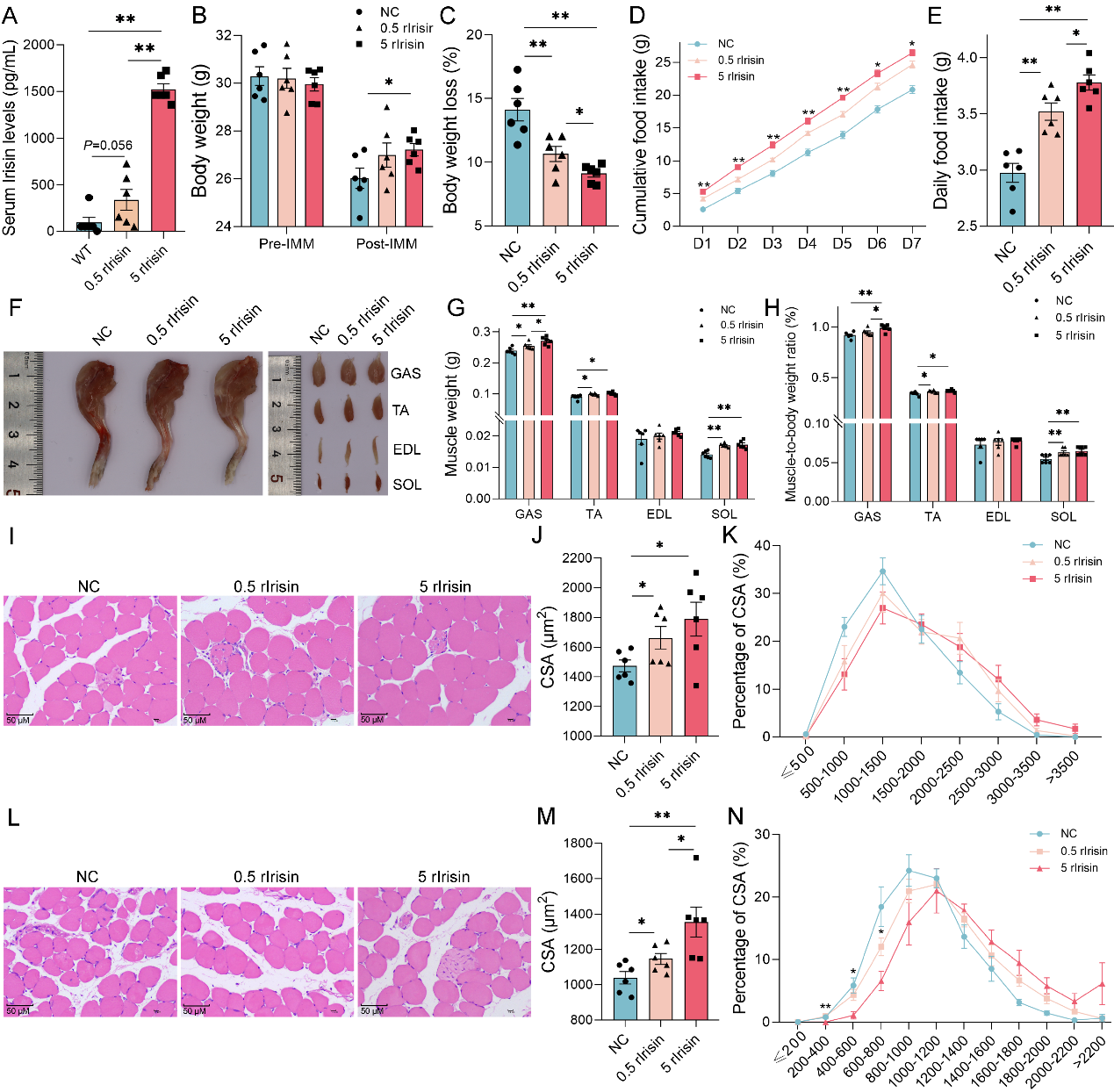


(A) Serum Irisin levels of mice daily receiving IP of 0.5 μg/mouse/week or 5 μg/mouse/week rIrisin. (B and C) Body weight changes (B) and body weight loss (C). (D and E) Quantification of cumulative food intake (D) and daily food intake (E). (F) Representative images of hindlimbs and GAS, TA, EDL, and SOL muscles. (G) Weight of GAS, TA, EDL, and SOL muscles. (H) Muscle-to-body weight ratios of GAS, TA, EDL and SOL muscles. (I-K) Representative H&E staining (I), mean myofiber CSA (J) and the percentage of CSA distribution (K) of TA muscles. (L-N) Representative H&E staining (L), mean myofiber CSA (M) and the percentage of CSA distribution (N) of SOL muscles.

Age and body weight-matched male adult OCN^-/-^ mice were under IMM condition for seven days and received daily intraperitoneal injection of rIrisin at different doses (0.5 μg/mouse/week or 5 μg/mouse/week). Thus, three groups of mice (NC, 0.5 rIrisin and 5 rIrisin) were studied here. Representative images (scale bar = 50 μm) captured at 400× magnification. n=6 for each group unless otherwise specified, data points show individual mice. Data are represented as mean ± SEM and were analyzed by one-way ANOVA with Tukey's post hoc test. * *p* < 0.05.** *p* < 0.01. The * in Fig. C and M mean significant difference between 0.5 rIrisin and 5 rIrisin.

**Fig. S11 FNDC5 is differentially expressed in porcine skeletal muscle at different ages**


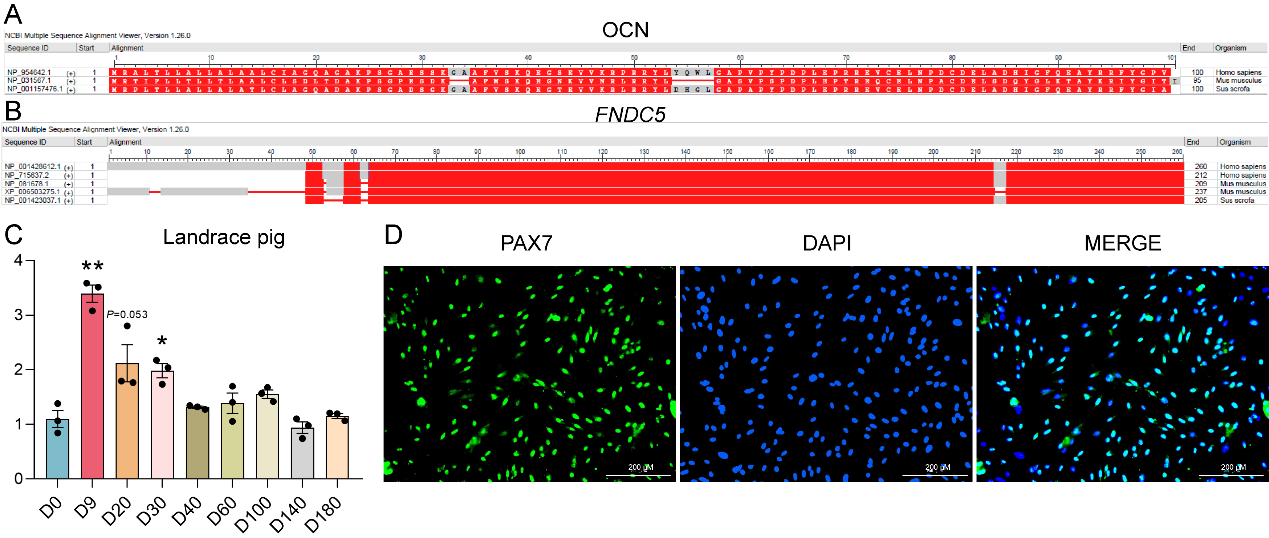


(A and B) The sequence conservation of OCN (BGLAP1/2) (A) and FNDC5 (B) across mice, human, and pig. (C) Porcine FNDC5 level (FPKM) at different time-points (n = 3 for each group). (D) Representative immunofluorescence staining of PAX7 of porcine MSC (n = 3).

The sequence conservation of OCN (BGLAP1/2) and FNDC5 across mice, human, and pig were analyzed by National Center for Biotechnology Information (https://www.ncbi.nlm.nih.gov/gene/). The publicly available porcine muscle transcriptome data (GSE157044) were analyzed. Representative images (scale bar = 200 μm) captured at 10× magnification. Number of samples per group is specified in the corresponding figure legend, data points show individual sample. Data are represented as mean ± SEM and were analyzed by unpaired Two-tailed Student's *t* tests or one-way ANOVA followed by Dunnett's post hoc test. * *p* < 0.05.** *p* < 0.01.

**Supplementary Tables**

**Table S1 siRNA sequences**

| Name | species | Sequence (5'-3') | |
| --- | --- | --- | --- |
| si-Gprc6a-1 | Mouse | sense | GCUAGUGAUAAUUGGUCAA |
| si-Gprc6a-1 | Mouse | antisense | UUGACCAAUUAUCACUAGC |
| si-Gprc6a-2 | Mouse | sense | CACUGUUAUCCGGAGUCAA |
| si-Gprc6a-2 | Mouse | antisense | UUGACUCCGGAUAACAGUG |
| si-Gprc6a-3 | Mouse | sense | CGUGGAGAUUAUAGUCAUU |
| si-Gprc6a-3 | Mouse | antisense | AAUGACUAUAAUCUCCACG |
| si-Fndc5-1 | Mouse | sense | CAAUAACAACAAGGAGAAA |
| si-Fndc5-1 | Mouse | antisense | UUUCUCCUUGUUGUUAUUG |
| si-Fndc5-2 | Mouse | sense | CCUCAAAGAACAAAGAUGA |
| si-Fndc5-2 | Mouse | antisense | UCAUCUUUGUUCUUUGAGG |
| si-Fndc5-3 | Mouse | sense | CCAUCUCUCAGCAGAAGAA |
| si-Fndc5-3 | Mouse | antisense | UUCUUCUGCUGAGAGAUGG |
| si-FNDC5 | Pig | sense | CCCAAUAAUAGCAAAGAAA |
| si-FNDC5 | Pig | antisense | UUUCUUUGCUAUUAUUGGG |
| Piezo1 | mouse |  | UCGGCGCUUGCUAGAACUUCA |

**Table S2 primer sequences**

| Name | species | Sequence (5'-3') |
| --- | --- | --- |
| Gapdh | Mouse | F: GCTGAGTATGTCGTGGAGT |
| Gapdh | Mouse | R: GTTCACACCCATCACAAAC |
| Bglap1/2 | Mouse | F: GGAGGGCAATAAGGTAGTGAAC |
| Bglap1/2 | Mouse | R: CAAGCCATACTGGTCTGATAGC |
| Piezo1 | Mouse | F: CGCTGTGTACACCTTCCAGT |
| Piezo1 | Mouse | R: TCCGACACACTGAACTGCTC |
| Fndc5 | Mouse | F: GAGGTGACCATGAAGGAGATG |
| Fndc5 | Mouse | R: GCGGCAGAAGAGAGCTATAA |
| Fbxo32 | Mouse | F: TCAAAGGCCTCACGATCACC |
| Fbxo32 | Mouse | R: TCAAACGCTTGCGAATCTGC |
| Trim63 | Mouse | F: GCTGAGTAACTGCATCTCCAT |
| Trim63 | Mouse | R: GCTATTCTCCTTGGTCACTCTG |
| Atp8b2 | Mouse | F: CACCACCATTGTGCCTTTGG |
| Atp8b2 | Mouse | R: CAGCACCTGAGAATGACGGT |
| Csnk2a2ip | Mouse | F: CACCACTACCCGACTGTAGC |
| Csnk2a2ip | Mouse | R: CGCGTGCTAGGATCACATCT |
| Casp12 | Mouse | F: ATTGTGAGAGCCACCCCTTC |
| Casp12 | Mouse | R: TGGGGAACCACCAGACCTTA |
| Trpm3 | Mouse | F: ACACAAGAATCGGTCAAGGGG |
| Trpm3 | Mouse | R: CCACTCCCATCACAGACCAC |
| GAPDH- | Pig | F: TCGGAGTGAACGGATTTGGC |
| GAPDH | Pig | R: TGCCGTGGGTGGAATCATAC |
| FNDC5 | Pig | F: CGGTGTCATTGCCCTCTTCT |
| FNDC5 | Pig | R: CAGCAAAGGCTTGATGGCAG |
| PIEZO1 | Pig | F: GTGTCCTCTCTGGCTGTCAC |
| PIEZO1 | Pig | R: CCTCAGCAGCTTCTCCTTCA |
| FBXO32 | Pig | F: GAGAAGAGTGGCAGCTTCGT |
| FBXO32 | Pig | R: TCTCTTCTTGGCCGCAACAT |
| TRIM63 | Pig | F: CATGTGCAAGGAGCACGAAG |
| TRIM63 | Pig | R: TGGAGATGCGGTTACTCAGC |

**Table S3 Primary and Secondary antibody**

| Name | Dilution | Cat No |
| --- | --- | --- |
| MyHC | 10 µg/mL | R&D-MAB4470-SP |
| Pax7 | 1:100 | Abcam- ab187339 |
| Cy3 conjugated Goat Anti-mouse IgG (H+L) | 1:200 | Servicebio-GB21301 (red) |
| Goat Anti-Mouse lgG (H+L) FITC | 1:200 | Abways-AB0122 (green) |
